# Computational Characterization of 3′ Splice Variants in the GFAP Isoform Family

**DOI:** 10.1371/journal.pone.0033565

**Published:** 2012-03-30

**Authors:** Sarah E. Boyd, Betina Nair, Sze Woei Ng, Jonathan M. Keith, Jacqueline M. Orian

**Affiliations:** 1 School of Mathematical Sciences, Monash University, Clayton, Victoria, Australia; 2 Department of Biochemistry, La Trobe University, Bundoora, Victoria, Australia; University of Edinburgh, United Kingdom

## Abstract

Glial fibrillary acidic protein (GFAP) is an intermediate filament (IF) protein specific to central nervous system (CNS) astrocytes. It has been the subject of intense interest due to its association with neurodegenerative diseases, and because of growing evidence that IF proteins not only modulate cellular structure, but also cellular function. Moreover, GFAP has a family of splicing isoforms apparently more complex than that of other CNS IF proteins, consistent with it possessing a range of functional and structural roles. The gene consists of 9 exons, and to date all isoforms associated with 3′ end splicing have been identified from modifications within intron 7, resulting in the generation of exon 7a (GFAPδ/ε) and 7b (GFAPκ). To better understand the nature and functional significance of variation in this region, we used a Bayesian multiple change-point approach to identify conserved regions. This is the first successful application of this method to a single gene – it has previously only been used in whole-genome analyses. We identified several highly or moderately conserved regions throughout the intron 7/7a/7b regions, including untranslated regions and regulatory features, consistent with the biology of GFAP. Several putative unconfirmed features were also identified, including a possible new isoform. We then integrated multiple computational analyses on both the DNA and protein sequences from the mouse, rat and human, showing that the major isoform, GFAPα, has highly conserved structure and features across the three species, whereas the minor isoforms GFAPδ/ε and GFAPκ have low conservation of structure and features at the distal 3′ end, both relative to each other and relative to GFAPα. The overall picture suggests distinct and tightly regulated functions for the 3′ end isoforms, consistent with complex astrocyte biology. The results illustrate a computational approach for characterising splicing isoform families, using both DNA and protein sequences.

## Introduction

Astrocytes are non-neural (or glial) cells and the most numerous cell type of the central nervous system (CNS) [1]–[3]. The recent literature has highlighted the remarkable diversity of interactions and functions that this cell type is capable of engaging in, probably not witnessed in any other mammalian cell [4], [5]. Currently, the consensus is that astrocytes participate in every aspect of brain activity under normal [6]–[12], injured and pathological conditions [13]–[16].

Generally, astrocytes are divided into two major groups, based on their localization and morphology, namely protoplasmic astrocytes predominantly in CNS grey matter, and fibrous astrocytes predominantly in white matter [17]. Superimposed on this classification is another grouping that recognizes additional subtypes (up to 9 in primates), based on further structural specialization, contacts and complexity of branching of processes [1], [17], [18]. Presumably, this variability in morphology underlies the wide-ranging interactions that this cell-type is capable of, in order to perform the multiplicity of functions attributed to it.

*In vivo* investigation of astrocytes has been challenging, and classical approaches such as analysis of astrocytic markers by epifluorescence or confocal microscopy have remained essential tools for improved understanding of astrocyte function. To this effect, the cytoskeletal component glial fibrillary acidic protein (GFAP) [19], [20] has been the preferred marker, presumably due to a number of factors. First, it is widely expressed across astrocyte sub-types [19], [20]. Second, well-established methods are available for the large-scale purification of this polypeptide, which has resulted in extensive characterization of its biochemical properties [19]. Third, it is highly antigenic and a number of reliable commercial antibody preparations are obtainable for this protein [21], [22]. GFAP is an intermediate filament (IF) protein of 49.9 kDa, [19], [20]. It has been extensively investigated in humans and other species, particularly rodents, because of reactive changes (or gliosis) in astrocytes associated with ageing [23], [24], following insult/injury [25], [26] and in neurodegenerative disorders such as Alzheimer's and Alexander's diseases [27]–[29], as well as multiple sclerosis [30], [31]. In addition, GFAP has also been associated with other tissues containing cells of stellate morphology, such as the pancreatic and hepatic stellate cells [32]–[34] and enteric glia [35].

**Figure 1:**
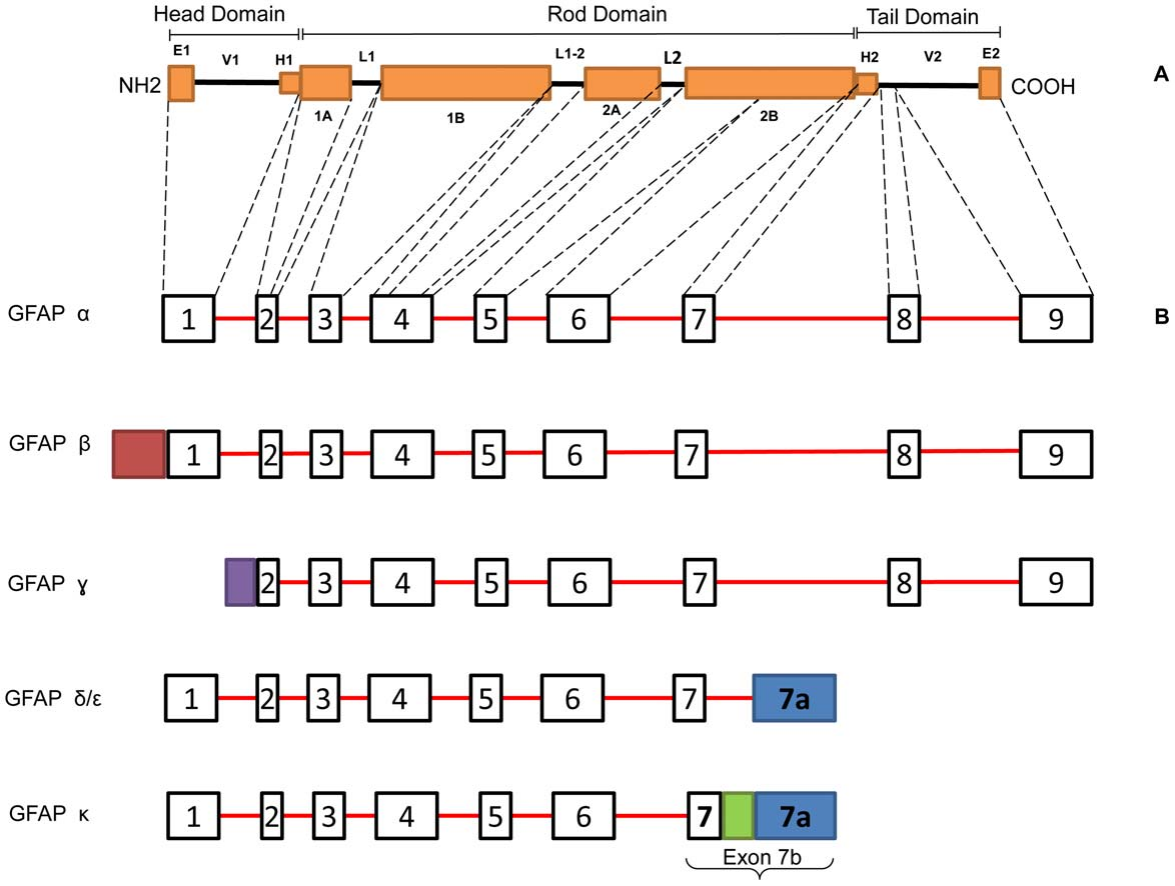
The GFAP family of isoforms. **A.** The secondary structure of the GFAP major isoform has the typical organization of IF proteins. It consists of an α-helical central rod domain flanked by the amino terminal head and carboxy terminal tail domain. The central rod domain consists of four subdomains, namely 1A, 1B, 2A and 2B interrupted by linker regions L1, L1–2 and L2. The head and tail regions are also divided into the highly charged E1 and E2 domains, variable domains V1 and V2 and the hypervariable stretches H1 and H2. **B.** At least 4 additional isoforms have been described, resulting from alternative splicing at the 5′ or 3′ end. Exons are shown as black boxes and introns as red lines. The relationship between exons and subregions is shown by dotted lines. *GFAPα* is the major isoform and contains all 9 exons. *GFAPβ* has an upstream transcriptional start sequence relative to GFAPα, resulting in additional 5′ mRNA sequences (red box), but the same protein sequence. *GFAPγ* lacks exon 1 and part of intron 1, but has a transcriptional start site within exon 1 resulting in an altered 5′ mRNA sequence (purple box); however, its translational start site is still undetermined. *GFAPδ* (rat) and *GFAPε* (mouse) appear to be the same product and lack exons 8 and 9 but contain exon 7a (blue box) derived from alternative splicing of intron 7. *GFAPκ* also lacks exons 8 and 9, but contain a novel exon 7b, comprised of exon 7, a proximal region of intron 7 (green box) and exon 7a.

GFAP occurs as a family of isoforms with splice variants at both the 5′ and 3′ end. To date 6 isoforms have been described from normal human and rodent sources [28], [36]–[41] (Fig. 1). GFAPα was the first identified, from multiple sclerosis plaques, and is by far the most abundant [19]. GFAPβ, isolated from the rat RT4-D6 Schwann cell line, has a translation initiation site upstream of GFAPα, but the two final polypeptides are identical [37], [38]. GFAPγ, first identified from mouse bone marrow and spleen, lacks exon 1 and part of intron 1; however the start signal of this isoform is still unidentified and its protein sequence is unpublished [40]. GFAPδ, from primary astroglial cultures of newborn rats [41], GFAPε, from human brain, [39] and GFAPκ, from human high grade gliomas and mouse and pig brains [36], differ from GFAPα at the 3′ end, by the absence of exons 8 and 9 in the transcripts and alternative splicing of exon 7 and intron 7 [20]. The existence of distinct δ and ε forms is controversial and GFAPδ and GFAPε appear to give rise to the same product [20], [42]. In addition a further 3 isoforms have been associated with Alzheimer's disease, Down syndrome and epilepsy [28], [43].

The GFAP sequence can be divided into the three major domains typical of IF proteins: the amino terminal head region, an α-helical rod domain and the carboxy terminal tail region (Fig. 1). The typical sequence of events in IF assembly is as follows. The first step is the formation of parallel and anti-parallel dimers by the α-helical rod domains of two polypeptides. These readily associate to form stable tetramers or protofilaments. This is followed by the association of two protofilaments to form a protofibril which gives rise to the 10 nm IF [44]. Each of the domains plays a role in the complex assembly process. The head domain is critical for filament assembly and regulation of end-to-end interactions. The highly conserved rod domain is overall hydrophobic and plays a crucial role in filament assembly formation, by facilitating coiling between IF polypeptides [35]. Likewise, the tail domain plays a role in the stabilization of the IF by facilitating lateral interactions at the protofilament/protofibril level, thereby influencing filament diameter [44], [45].

The observation of alternative splicing in IF proteins, including GFAP, has been documented, but is puzzling because of the potential consequences on assembly of the cytoskeleton. The variation in the tail domain of splice variants should have a critical effect on cytoskeletal assembly. Investigations of structure-function relationships for GFAP isoforms are few, but informative. In vitro studies of GFAPδ/ε and GFAPκ show that although they cannot form homomeric filaments, they are capable of forming heteromeric structures with GFAPα [46]. These heteromeric filaments appear to be associated with the cytoskeleton as well as other complex molecules/structures, which suggests that these minor isoforms are required for additional functions within the context of the cytoskeleton [30]. There is also increasing evidence that IF proteins are important modulators of cellular function [31], [43]. The variety of GFAP isoforms, the extensive diversity of astrocyte interactions and functions, and the implication of GFAP in CNS function and neurodegenerative diseases, all suggest that GFAP may have a more diverse and fundamental role than cytoskeletal assembly alone, particularly through the minor isoforms.

Better characterisation of the GFAP isoforms is highly important for CNS research, to gain more insight in the modulation of astrocyte function under normal homeostatic conditions or neurodegenerative disease. Here we focus on the isoforms resulting from variation in the exon 7/intron 7 region. To identify the unique features of these isoforms and the mechanisms by which they are regulated, we used a variety of computational methods to analyse both the DNA and protein sequences of GFAPα, GFAPδ/ε and GFAPκ in mouse, rat and human. Jointly considering both DNA and protein in this manner is not common practice, but we find that each illuminates the other. We identified structural and functional features that define the different isoforms, and found evidence of an as yet unidentified isoform. The results at both the DNA and protein level suggest that generation of the GFAP isoforms is tightly regulated through conserved mechanisms to produce biologically diverse products consistent with the diverse morphology and function of astrocytes.

## Results

### DNA Sequence Conservation

Multiple change-point analysis can be used to identify changes within biological sequences based on a specific property, for example amino acid or codon usage. In genomics, it has been particularly successful at identifying intra- and inter-species patterns of conservation in DNA [47], [48], and is currently being applied to identify putative functional classes including protein-coding and regulatory sequences. This method has so far only been used for large-scale analysis (whole genomes or chromosomes). We investigated the utility of this approach for analysing much shorter DNA sequences, in particular alternative splicing of a single gene. Our expectation is that working with shorter sequences will not significantly change the sensitivity of the method to the presence of a change-point, since these are essentially local features of the sequence. However, we anticipate that the ability to discriminate between distinct classes of conservation level will be greatly reduced when working with shorter sequences, because fewer segments from each class are available.

**Figure 2:**
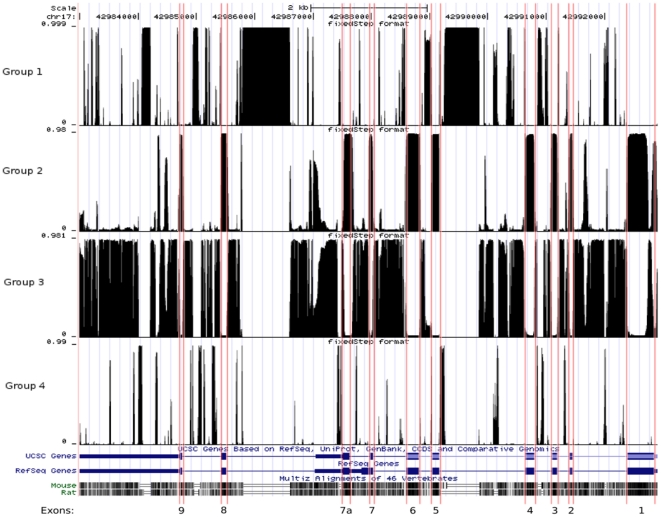
The four segment classes identified in the GFAP gene using changept. The top four profiles show, for each sequence position in the human GFAP DNA sequence (chr17: 42982993–42992914 in UCSC genomic coordinates), the probability that the base at that position belongs to conservation groups 1 to 4 respectively, as identified by the program changept applied to a 3-way alignment of rat, mouse and human sequences. At any position, the sum of the four profiles is 1. The two rows below the Group 4 profile display the exons (wide bars), the UTRs (narrow bars) and the introns (thin lines) of GFAP genes recorded in the UCSC and RefSeq collections respectively. Below these are the UCSC conservation tracks relative to mouse and rat, in which darker regions correspond to higher conservation, and parallel lines indicate deletions. At the bottom of the figure are the exon numbers. Note that the gene is transcribed from right to left. Exon boundaries are indicated with red vertical lines. *Group 1* identifies regions of insertions specific to the human version of the gene; *group 2* corresponds mainly to the mapped exons of the GFAP gene, appearing to cover regions of high conservation between the three species; *group 3* is comprised of segments in which deletions occur in either the rat or the mouse genes, but not both; *group 4* represents the least conserved parts of the gene.

We generated a 3-way alignment of human, rat and mouse DNA sequences, using a 200 kB fragment of DNA centered on GFAP, and used the software changept [47], [48] to search for classes of conservation that captured features of structure and functional regulation of GFAP. Only three species were considered because alternative splicing variants have been extensively studied in only these species. We identified four classes of segment that were characterised by distinct patterns of conservation (Figure 2). We investigated the characteristics of the alignment columns that could be unambiguously assigned to each class (Figure S1, Table S1). One of the segment classes (Group 1) corresponded to regions where there are insertions specific to the human version of the gene, and a second class (Group 4) was comprised of segments in which deletions occur in either the rat or the mouse genes, but only rarely in both. Both of these segment classes are predominantly intronic, but are also observed in the 3′ UTR of the gene. They may represent regions that are functionally unimportant and can be deleted without detriment to the organism. Alternatively, they may have lineage-specific functional roles. The fact that there are extensive human-specific insertions, but no corresponding mouse- or rat-specific insertions, perhaps favours the latter interpretation. A third segment class (Group 2) is of most interest, corresponding remarkably closely with the mapped exons of the GFAP gene, and appearing to cover regions of high conservation between the three species. The fourth class (Group 3) makes up the balance, representing the less well-conserved parts of the gene. A curious feature of Group 3 is that it contains a high proportion of alignment columns in which rat and mouse match but human differs. Throughout the rest of this paper, we refer to regions of the gene with a high Group 2 profile value as *conserved regions*, and they form the focus of the remainder of our analysis. We also focus here on the features related to alternative splicing at the 3′ end of GFAP, even though there are several conserved regions throughout the 5′ end of the sequence.

**Figure 3:**
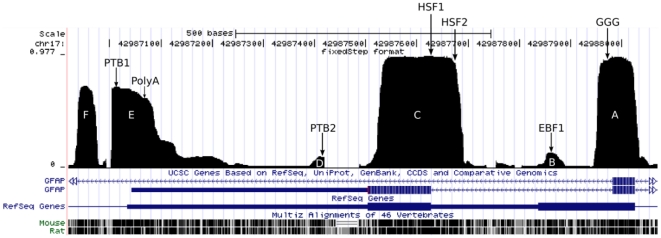
Conserved features across exon 7/7a/7b of GFAP. The profile shows detail of the Group 2 profile from Figure 2 in the region surrounding exons 7 (right of screen) and 7a (right of centre). Exons (wide bars), UTRs (narrow bars) and introns (arrowed lines) are shown for two genes in the UCSC collection and one in RefSeq. At the bottom is the UCSC conservation profile relative to mouse and rat. Conserved regions are labelled A–F (in white). Labelling is from right to left to match the order in which exons are displayed. Conserved motifs that were identified are labelled as follows: GGG, the 3G triplets (feature A); EBF1, motif recognized by the transcription factor EBF1 (feature B); HSF1 and HSF2, the actual and possible acceptor sites identified by Human Splice Finder (scores 93.19 and 76.63 respectively, feature C); PTB1 (feature D) and PTB2 (feature C), conserved PTB-binding motifs embedded in polypyrimidine-rich sequences; PolyA polyadenylation signal AAUAAA (feature E). All other symbols are as per Figure 2.

Most of the conserved regions are known to code for proteins. In fact, if we regard alignment columns with a Group 2 profile greater than 0.5 as putatively protein coding, then we can estimate the sensitivity and specificity of the resulting classifier to be 0.867 and 0.937 respectively (see Methods). In general, the start and end points of the conserved features occur at or very close to the boundaries of the exons (Figure 2). In the case of those features in the neighbourhood of exons 7 and 7a, however, there are some relevant departures from this rule (Figure 3; labelled conserved features A–F). Conserved feature A does not terminate immediately after exon 7, but instead extends for ∼30 nucleotides into intron 7. There is a region ∼60 nucleotides further downstream with low but slightly elevated conservation (B). Following the pattern of conserved feature A, feature C begins ∼50 nucleotides upstream of the start of exon 7a, and is followed by a small feature (D) further downstream. At the 3′ end of the 3′ UTR of the short isoforms, feature E does not have the mesa-like appearance of most Group 2 regions, suggesting that it may contain several distinct conservation features that the segmentation analysis was unable to resolve. It may also be linked to feature F, which is located approximately 65 bases downstream of this 3′ UTR.

Conserved features B and D require special mention, as they have only slightly elevated Group 2 profile values, and indeed have much higher Group 3 profiles (Figures S2 and S4, Tables S2 and S4). We compared the alignment column patterns for these features (Figures S3 and S5, Tables S3 and S5) and found that the proportion of 3-way match columns (AAA) is similar to that of Group 2, whereas the proportion of 2-way match columns (AAB, ABA and ABB) is similar to that of Group 3. The short length of these features may also have played a role in confusing changept.

### DNA Regulatory Features

Conserved feature B is located at the very end of the last GFAPκ exon (Figure 3), suggesting the possible presence of one or more regulatory elements. The TOMTOM motif comparison tool [49] was used to search the human sequence of feature B for any known motifs. One significant motif was identified: a putative binding site for the transcription factor early B-cell factor 1 (EBF1; Figure S6). However, feature B is an inappropriate location for transcription factor binding, unless there exists an undiscovered short isoform with transcription initiation site upstream. As neither the mouse nor rat sequences had any significant TOMTOM hits in this region, we did not pursue this possibility further. BLAST was also used to search NCBI for sequences similar to feature B, as a simple method for checking whether the region contains an as yet unidentified motif, but no significant results were returned. Nevertheless, the location of feature B remains suggestive of a regulatory role.

Conserved feature C has an extended region that has not been reported as protein-coding, but is confidently predicted as belonging to the same conservation class that includes all the other exons, and has the striking mesa-like appearance of the other exons. To search for possible novel splicing sites, we submitted a 464 nt human sequence spanning GFAP exons 7 and 7a to the Human Splicing Finder server (http://www.umd.be/HSF/HSF.html). Interestingly, there is a potential acceptor site (score 76.63 out of a possible 100) located 40 nt upstream of the conserved region, which is consistent with the hypothesis that this is a novel splice variant (Figure S7).

A fundamental mechanism of alternative splicing control is through splicing regulatory elements (SREs), which are identified as motifs in the mRNA sequence [50]–[53]. Control of alternative splicing by SREs is highly complex and is the focus of ongoing research, however there are some well-known SREs that are relevant to astrocytes include G triplets, CA repeats, the YCAY motif (recognised by the Nova family of proteins), and the CUCUCU and UCUUC motifs (recognised by polypyrimidine tract binding protein, or PTB) [50]–[52], [54], [55]. Although very little is known about how alternative splicing of GFAP is regulated, the expression levels of PTB have been demonstrated to affect the ratios of the different GFAP isoforms by an unknown mechanism [36]. Using simple Perl scripts to execute pattern search, we searched for occurrences of these four SRE motifs, looking for clusters of G runs, CA repeats and YCAY motifs, as well as occurrences of the PTB motif (not necessarily clustered) that were conserved across all three species and situated at locations relevant to the generation of the 3′ GFAP isoforms. We identified two PTB motifs, both of which were located in conserved regions, specifically features D and E (Figure 3).

### Protein Sequence Conservation

Conservation of protein sequences amongst multiple species suggests that the sequence has been maintained throughout evolution despite speciation, which in turn implies functional importance of the protein, or the region of protein that is conserved. The complete protein sequences for GFAPα and GFAPδ/ε for the three species of interest have been determined. The protein sequence for GFAPκ was derived from cDNA as described [36]. The amino acid sequences of the GFAPα, GFAPδ/ε and GFAPκ isoforms are almost entirely conserved - in the head and rod domains, but then vary in the C-terminal tail domain (Table 1). In all three species, the length of the GFAPα tail is essentially conserved, with just a single additional residue in the mouse protein. The length of the GFAPδ/ε isoform tail is the same in human and mouse, but shortened in rat. The tail region of GFAPκ is also similar in length in human and mouse, but in rat the tail is greatly truncated.

**Table 1:** Comparison of the head, rod and tail domain sequences of GFAP isoforms across species.

Species	GFAP isoform	Exon usage for each isoform	Total length of polypeptide [length of head+rod domains] (number of aa)	Length of tail domain [length of variable region] (number of aa)
Mouse	GFAP α	1–7	430 [374]	56
	GFAP δ/ε	1–7, 7a	428 [374]	54 [43]
	GFAP κ	1–6, 7b	435 [374]	61 [55]
Human	GFAP α	1–7	432 [377]	55
	GFAP δ/ε	1–7, 7a	431 [377]	54 [42]
	GFAP κ	1–6, 7b	438 [377]	61 [48]
Rat	GFAP α	1–7	430 [375]	55
	GFAP δ/ε	1–7, 7a	421 [375]	46 [42]
	GFAP κ	1–6, 7b	409 [375]	34 [20]

For each species, the exon usage for isoforms with 3′end splice variation is shown, relative to the major isoform GFAPα. For each isoform, the total length of the polypeptide is shown in terms of number of aa, followed by the length of the combined head+rod domains in brackets (i.e encoded by exons 1–6, see Figure 1B). The tail domain consists of exons 7–9 in GFAPα, exons 7 and 7a in GFAPδ/ε and exon 7b (which includes exon 7, intron 7a and exon 7a, see Figure 1B, [36]) in GFAPκ. The length of the complete tail domain is shown for each isoform and the length of the variable regions for GFAPδ/ε and GFAPκ in brackets. The above data were generated from the UniProt Knowledgebase database.

**Figure 4:**
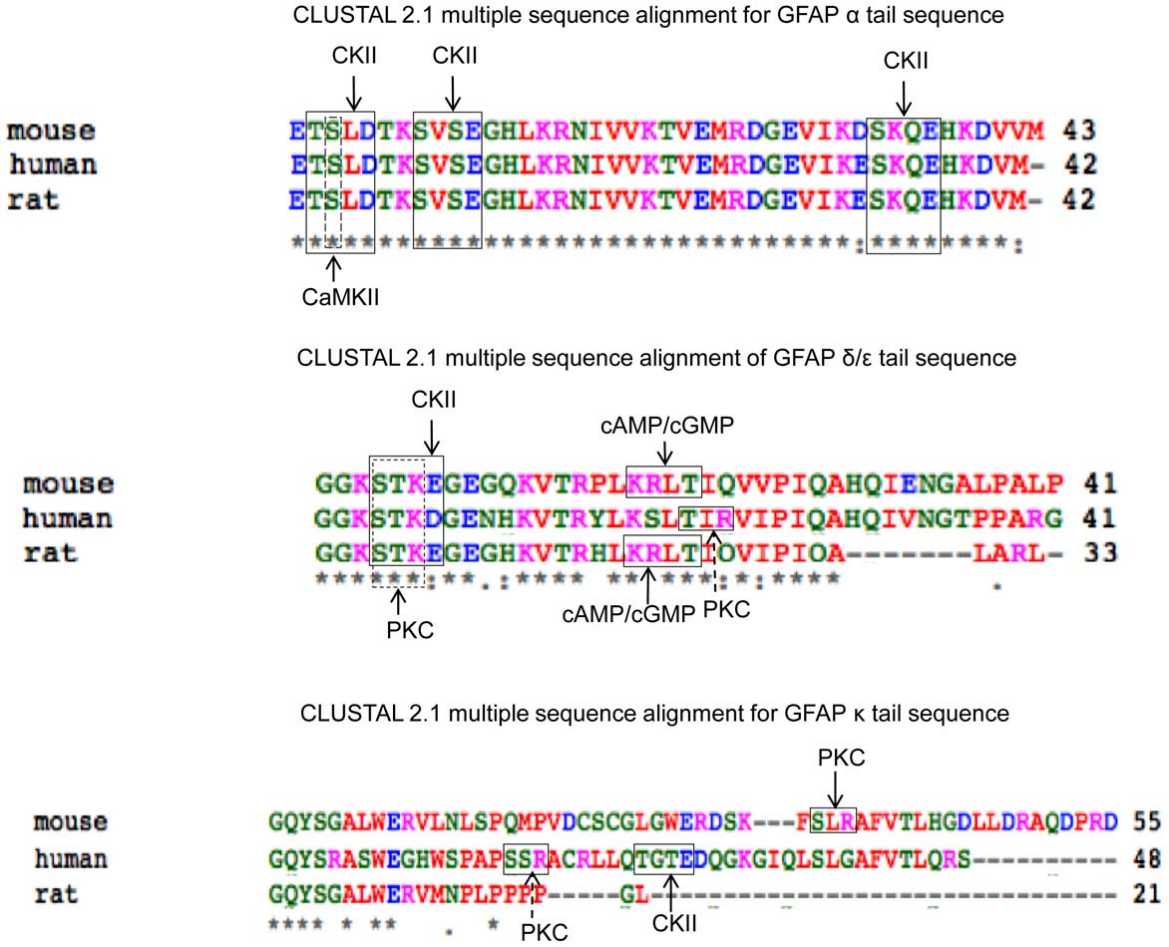
Comparison of human, rat and mouse sequences for GFAPα, GFAPδ/ε and GFAPκ. Sequences for GFAPα, GFAPδ and GFAPε for the three species and GFAPκ for human and rat were obtained from the NCBI database. Mouse GFAPκ was reconstructed from the genomic sequence and cross-referenced with the sequence reported in Bleichenberg et al (2007). Clustal W results from TCoffee server and predicted phosphorylation sites from PROSCAN are shown. Predicted recognition sites are highlighted by rectangles and predicted phosphorylation sites by an arrow. The phosphorylation site by CaMKII in GFAPα (as annotated in NCIB and HPRD) is also shown. Amino acids (aa) are represented as follows: RED = small aa (small+hydrophobic (incl.aromatic -Y)); BLUE = acidic; MAGENTA = basic; GREEN = hydroxyl+sulfhydryl+amine+G; Grey = unusual amino/imino acids.

Cross-species conservation of the amino acids in the tail regions of the isoforms was assessed using ClustalW2 (Figure 4; (http://www.ebi.ac.uk/Tools/msa/clustalw2/) [56], [57]. The GFAPα tail sequences are very highly conserved, being identical between human and rat, while in mouse there is a conservative E→D (glutamate to aspartate) substitution at residue 420, and an additional V (valine) residue inserted right before the C-terminal M (methionine). GFAPδ/ε is also well conserved between the species, particularly for the first 29 residues, from which point the rat isoform appears to have a deletion of residues relative to the mouse and human sequences. The GFAPκ isoform has poor conservation, in part due to the truncation of the rat tail sequence, but even between the human and mouse isoforms, there is initial conservation that rapidly breaks down after the first ∼10 residues in the tail.

### Phosphorylation sites

Phosphorylation is a fundamental mechanism for regulating protein activity and as expected, GFAP contains conserved phosphorylation sites. The NCBI Protein database and the Human Protein Reference Database[58] both report a single confirmed phosphorylation site in the tail of human GFAPα by CaM kinase II, which we identified as being conserved in mouse and rat. We therefore used the PROSCAN software on the Network Protein Sequence (NPS) Analysis server (http://npsa-pbil.ibcp.fr/cgi-bin/npsa_automat.pl?page=/NPSA/npsa_proscan.html), to search for putative phosphorylation sites with 100% similarity, i.e. no mismatch (Figure 4).

Three possible Casein Kinase II (CKII) sites were identified in the GFAPα tail, with the acceptor site motif [ST]XX[DE]. As expected, given the conservation of this tail sequence, these sites are predicted in all three species. CKII phosphorylation rate is increased by the presence of an acidic residue at the N-terminal of the acceptor site, which is the case for both the first (‘TSLD’) and third (‘SKQE’) predicted sites. In rat, the N-terminal residue for the third acceptor site is a D, as opposed to E in both mouse and human. If this were a true phosphorylation site, it is interesting to note that this conservative substitution would preserve the positive influence on phosphorylation rate. The second predicted site (‘SVSE’) has a basic residue (K) at the N-terminal, which decreases phosphorylation by CKII.

In GFAPδ/ε, there are two overlapping acceptor motifs in the N-terminal of the tail region, one for PKC (‘STE’) with acceptor site [ST]X[RK], and again one for CKII (‘STKE’), but both of them predict phosphorylation of the same S (serine) residue. The PKC site has a basic K (lysine) residue at the N-terminal, which can enhance both the *V_max_* (maximal velocity) and *K_m_* (substrate concentration at half the maximal velocity) of the phosphorylation reaction. In the CKII site, an N-terminal K (lysine) residue is likely to decrease the reaction, but an acidic E (glutamate) at +5 from the acceptor site could increase the rate of phosphorylation. In the mouse and rat sequences, a cAMP/cGMP-dependent protein kinase phosphorylation site is identified with motif [RK]XX[ST] (‘KRLT’). This site is not present in the human sequence, and instead a PKC site is predicted that would result in phosphorylation of the same T (threonine) residue.

In the least-conserved GFAPκ sequence, there are no common predicted phosphorylation sites across the species. A PKC acceptor site is identified in the mouse sequence, and in the human sequence, CKII and PKC sites are identified. The truncated rat sequence does not contain any predicted phosphorylation sites.

### Structural analysis

Knowledge of the three-dimensional structure of a protein can reveal insight into its function. However, the Protein Data Bank (PDB) [55] does not currently contain any solved structures for any of the GFAP isoforms. A search for homologous structures in PDB was performed using PSI-BLAST on the NCBI server (http://blast.ncbi.nlm.nih.gov/Blast.cgi), which did not yield any significant results for the tail region, and a search of ModBase (http://modbase.compbio.ucsf.edu/modbase-cgi/index.cgi) [59] did not find any reliable structure models for the tail region either (data not shown).

**Figure 5:**
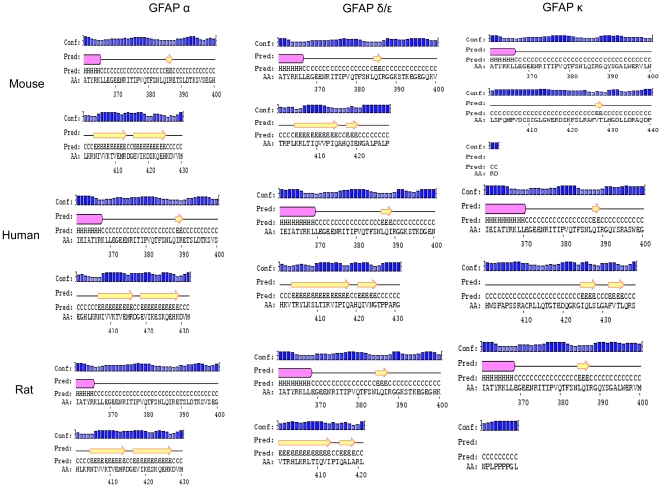
Comparison of secondary structures for the GFAP tail regions for the GFAPα, GFAPδ/ε and GFAPκ isoforms across species. The secondary structures of GFAP isoforms across human, mouse and rat were predicted using the PSIPRED server. Only the tail sequences were used as the sequences are similar up to exon 6. The program predicts the possibility of a helix (pink box), strand (yellow arrow) or a coil for the target amino acid.

In the absence of solved structures, the PSIPRED v3.0 server (http://bioinf.cs.ucl.ac.uk/psipred/) [60] was used to predict the secondary structure of the GFAP tail regions, using the default settings (Figure 5). For each of the three species, the tail of GFAPα is predicted to contain two long strand regions. For GFAPδ/ε, PSIPRED predicts a long strand followed by a shorter strand. This is consistent for GFAPδ/ε in all three species, however the first of the two strands is longest in human, and is shortest in mouse.

Consistent with the low conservation of the GFAPκ tail sequence, the predicted secondary structure of this isoform shows the most variation. Human GFAPκ has two very short strands predicted at the C-terminus, after a long coil region. This is similar to mouse, where the very short strand is predicted near the C-terminus. In mouse, a 2-residue strand that is predicted near residue 385 in both the α and δ/ε isoforms is not predicted in the tail of mouse GFAPκ. In rat, there is no predicted strand, only coil, but this is expected since the tail sequence of this isoform is significantly truncated relative to the other species. Variation in the tail sequence also affects where the predicted helix terminates (located at the boundary of the conserved and varying protein sequence).

### Hydrophobicity analysis

Hydrophobicity scales can be used to map a protein sequence for regions that are hydrophobic and hydrophilic. These regions can be used for structural inference, where hydrophobic regions tend to be found at the interior of a protein and hydrophilic regions tend to be found at the surface of the protein. For functional inference, highly hydrophobic surfaces imply regions of protein-protein interaction or association with lipid membranes, and hydrophilic properties suggest that the protein can move freely through aqueous environments including the cytoplasm. Therefore, similarly to the conservation of sequence and phosphorylation sites, the conservation of hydrophobic and hydrophilic regions suggests an additional level of structure and function that needs to be preserved.

**Figure 6:**
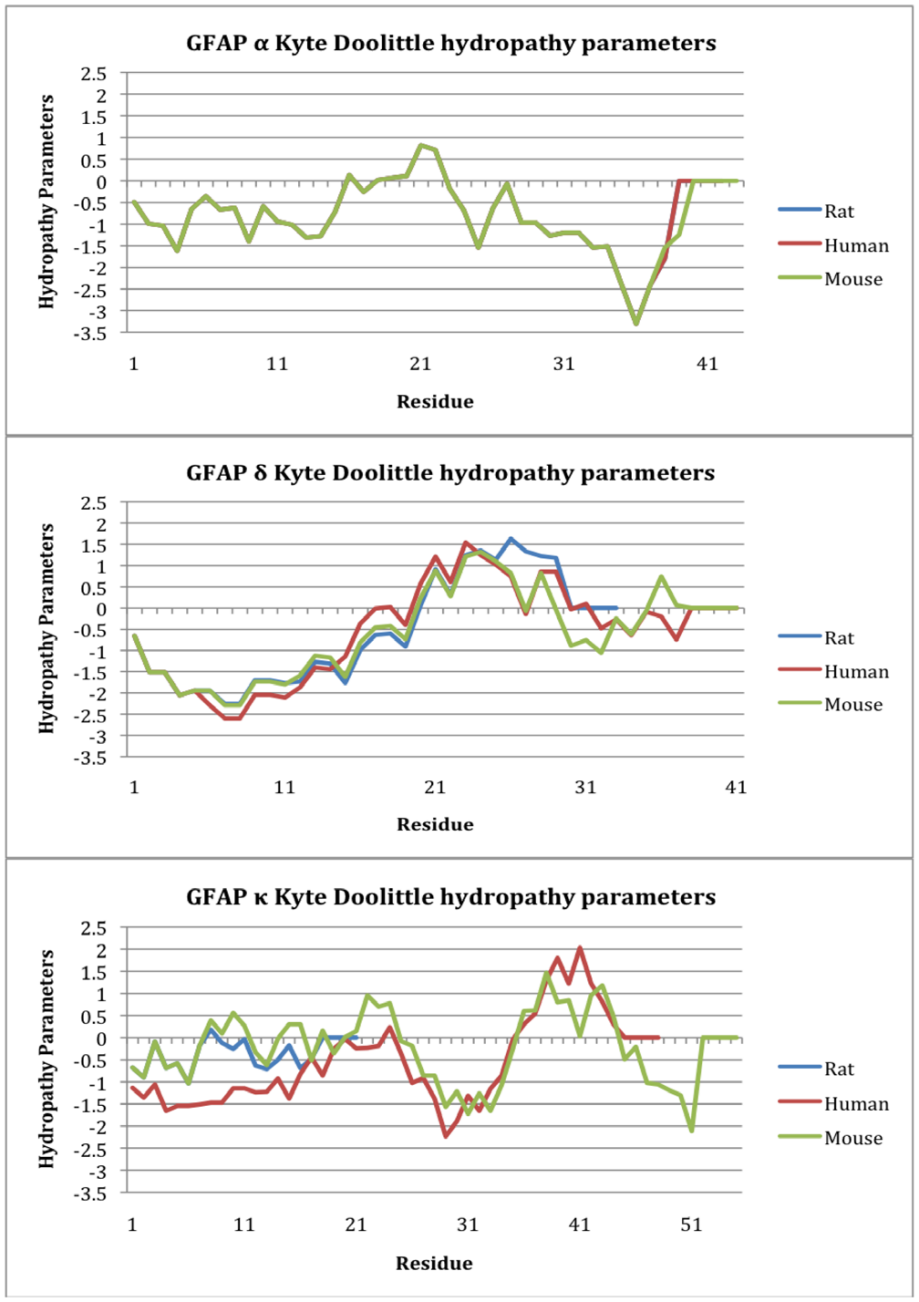
Kyte-Doolittle hydropathy plots for the tail regions of GFAP isoforms across the human, rat and mouse. The human, rat and mouse sequences are indicated by red, blue and green trendlines respectively. The peaks below zero represent hydrophilicity, whereas those above zero represent hydrophobicity.

Numerous hydrophobicity scales have been proposed, but a commonly used measure is the Kyte-Doolittle scale [61], which was developed from a wide range of published experimental observations. The EMBOSS Pepinfo tool (http://www.ebi.ac.uk/Tools/emboss/pepinfo/index.html) was used with a window width of 9 to generate hydrophobicity profiles for the different isoforms, which were compared between isoforms across the species (Figure 6). For comparison, the Optimal Matching Hydrophobicities (OMH) scale [62] was also used to generate hydrophobicity profiles. The OMH scale was developed using evolutionarily conserved structure and therefore can be considered to reflect size as well as hydrophobicity, making it relevant to considerations of protein-protein interactions [53]. The profiles generated from these two analyses are in agreement, so only the results from the Kyte-Doolittle scale are shown (Figure 6).

The highly conserved GFAPα tail sequences are generally hydrophilic. The GFAPδ/ε sequences are initially more hydrophobic, but where the GFAPα sequences have a slight trend towards a hydrophobic nature in the middle of the tail region, the GFAPδ/ε tails have a more pronounced hydrophobic stretch, and a less hydrophilic C-terminal (which is truncated in the rat sequence). The conservation of the GFAPκ tail regions is poor across the three species, but the hydropathy profiles show some surprising similarities. Although the rat GFAPκ sequence is greatly truncated relative to the mouse and human sequences, it has a hydropathy profile that is highly similar to the initial sequence of mouse GFAPκ. The mouse and human tails also have comparable hydropathy profiles. The human sequence is initially more hydrophilic than mouse (and rat), after which the mouse and human sequences follow a highly similar hydropathy profile (the rat sequence is missing in this region). At the C-terminus, the mouse sequence has a short stretch of hydrophilic residues that are, in comparison, missing from the human sequence.

## Discussion

Over the last decade the appreciation of the role of IF proteins has evolved. Originally believed to serve as elements forming cytoplasmic and nuclear networks providing cells with mechanical strength and specific moprphology, they are now known to facilitate essential non-mechanical roles, including for example, the regulation of signalling pathways and vectorial processes [15], [16], [26]. Presumably the occurrence of isoforms of IF proteins facilitates these alternative functions or adaptations, as it is likely that only limited variation in sequence can be tolerated in the elaboration of cytoskeletal architecture. The existence of a complex isoform family for GFAP is in agreement with evidence for (a) a multiplicity of inter-cellular interactions between this cell-type with other astrocytes, other glial cell types, neurons, as well as other structural CNS elements, such as blood vessels and (b) the remarkable morphological changes observed in this cell-type in response to insult to the CNS [52]. The limited number of studies relating to possible functions of GFAP isoforms demonstrate that they are generally of low abundance, but potentially capable of forming associations with other protein complexes. There have been reports of association of GFAPα with plectin [63], [64] and 14-3-3 a family of regulatory molecules that modulate IF structure [65], [66]. GFAPδ/ε has been shown to be associated with the heat-shock proteins αB-crystallin and Hsp 27 [67]–[70]. GFAPα/GFAPδ heteromers may be associated with changes in assembly properties of filaments and are observed around the nucleus of SW-13cl.1 cells [42]. However, these latter data need to be interpreted with caution, because they were based on transfection experiments, using recombinant GFAP. Finally, association of GFAPδ/ε (when overexpressed in cultured N2A neuroblastoma cell lines) with presenilin, has been demonstrated [39]. These collective observations prompted us to perform a computational analysis of the DNA sequence of exon/intron 7, which appears to be a ‘hotspot’ for splice variations, together with comparisons of protein sequences of the resulting isoforms. Biologists do not commonly look to computational approaches to investigate isoform families, and traditional biochemical approaches tend to result in splicing isoforms being identified by chance discovery. Generally, the focus is also at either the DNA level or the protein level. However, both DNA and protein sequences serve to control structure and function of isoform families. There is striking variation in the GFAP sequences, both amongst the isoforms and between species. However, it is the conserved regions that will be of most interest with respect to function and regulation. Here, we have used a novel application of multiple change point analysis, and numerous standard bioinformatics approaches, to characterise distinctive conserved features of the GFAP 3′ splicing isoforms at both the DNA and protein levels.

Using multiple change-point analysis, we identified a segment class that encompasses GFAP coding regions, as well as a number of conserved regulatory elements that are consistent with the structure and regulation of the three isoforms generated from alternative 3′ splicing. The ∼30 nt conserved region downstream of exon 7 (Figure 3; the 3′ end of feature A) is part of the final protein-coding exon of GFAPκ (i.e. exon 7b), and hence the conservation in this region may have functional significance for the protein. This conservation is apparent at the protein level, with the first ∼10 residues of the GFAPκ tail being conserved, and sharing of similar hydrophobicity and secondary structure properties. Conservation does not extend to the exon 7b stop codon, which is consistent with the analysis of the protein sequence, where the remaining GFAPκ tail sequences differ across the 3 species in both their amino acid sequences and their physicochemical properties. An alternative explanation of this conserved region is that it contains regulatory elements, such as splice regulatory elements. For example, this region contains three G triplets (GGG; Figure 3), present in all 3 species, which could act as a putative intronic splicing enhancer in the context of the 5′ splice site of exon 7, or as a splicing silencer in the context of exon 7b [55].

Conserved feature B (Figure 3) is of interest because it occurs at the very end of the last GFAPκ exon. Any conservation in this region is not likely to be due to protein function, as neither the protein sequence nor the hydropathy profiles of the GFAPκ tail are conserved. We were unsuccessful in identifying a specific regulatory element that was consistent with the structure, function or regulation of GFAPκ, but it nevertheless seems likely that a regulatory sequence exists in this region.

The ∼50 nt upstream extension of conserved feature C (upstream of exon 7a; Figure 3) has not been reported as a protein-coding region in any GFAP isoform, yet is confidently classified into the same class as all the other reported exons. We speculate that there is either an as yet unknown splice variant that incorporates this upstream region of exon 7a, or else a conserved control signal that regulates the splicing of this exon. The Human Splicing Finder server predicts a potential acceptor site 40 nt upstream of the conserved region, supporting the hypothesis of a new splice variant, and the fact that the acceptor site is identified as ‘good but not best’ is consistent with this being a less commonly expressed splice variant. Our analysis also suggests other complexity in the isoform family, as a result of features at the 5′ end of the sequence (Figure 2), but these features are beyond the scope of this paper.

The conserved region spanning the end of the short transcript UTR (feature E, Figure 3) also contains some interesting features. It contains a conserved polyadenylation signal AAUAAA near the 3′ end of the short transcript UTR (Figure 3, PolyA), which has been confirmed as being used [36], [71]. There is also a second polyA signal further upstream that has also been shown to signal polyadenylation ([71]; not illustrated), however this second polyA signal is not present in all three species, and therefore would not account for the GFAPκ and GFAPδ/ε isoforms in rat and mouse. At the 3′ end of feature E, there is a conserved CUCUCU motif (Figure 3, PTB1), and further upstream a conserved UCUUC motif (Figure 2, PTB2), both of which are potential binding sites for the polypyrimidine tract binding protein (PTB). PTB controls alternative processing and translation of RNAs, promotes exon exclusion, and polyadenylation [72], and mediates global silencing of weak or otherwise highly regulated exons [58]. Overexpression of PTB has been shown to influence the relative expression levels of the GFAP isoforms by an as yet unknown mechanism [71]. Both of these potential sites are embedded in pyrimidine-rich regions, which is optimal for PTB binding [73]. The existence of two PTB binding sites in close proximity would also allow PTB to form a dimer, where multimerization has been shown to be an important mechanism of PTB regulation [58].

All the identified features occur within the same segment class, which appears to encompass highly conserved genetic regions, and we have identified multiple regulatory elements that are also highly conserved across the three species. These give a picture of tight regulation of the alternative splice forms at the 3′ end of GFAP, and imply functional importance that has been conserved throughout speciation. Also of note in this analysis is that although we identified some short conserved regulatory elements within longer non-conserved segments, multiple change-point analysis did not identify and classify all such regions with high confidence, even though such features may be biologically important. In particular, there appear to be conserved, biologically relevant regulatory elements within features B and D, even though conservation was only identified there with low confidence.

At the protein level, the tail region of GFAPα is highly conserved, leading to a consistent hydropathy profile and predicted secondary structure across the three species. In addition, there is a conserved CaMKII phosphorylation site that has been confirmed, and three predicted CKII phosphorylation sites, also conserved across the three species. Two of these CKII sites have N-terminal acidic residues that would enhance phosphorylation. GFAPα is by far the most abundant of the GFAP isoforms, and these conserved features of hydrophobicity, structure, and real and putative phosphorylation sites, together with the almost identical amino acid sequence across the three species, is consistent with the biological evidence that the tail region of GFAPα plays a fundamental role in astrocyte function.

The GFAPδ/ε isoform does not have the same overall conservation as GFAPα, but is well conserved over the first half of the tail region. The first ∼20 residues of GFAPδ/ε are more hydrophilic than in the GFAPα hydrophobicity profile, but then the short stretch of following hydrophobic residues is more hydrophobic than the similar region in the GFAPα profile. The predicted secondary structure is consistent across the three species, and the N-terminus of the tail contains conserved predicted CKII and PKC phosphorylation sites, both of which would result in phosphorylation of the same S residue. A predicted phosphorylation site at residue 21 (T) of the tail has a different putative kinase in human (PKC) compared to rat and mouse (cAMP/cGMP-dependent protein kinase), but would again result in phosphorylation of the same T residue. The 3′ splice variants of GFAP only vary in the tail region, and the fully conserved features of GFAPδ/ε are found in the first half of the tail of this isoform. GFAPδ/ε has been found to be located around the nuclear envelope, suggesting that this specific localisation may be attributed to structural, physicochemical and regulatory features that are contained within these initial ∼20 amino acids of the tail sequence.

In contrast to these two isoforms, GFAPκ appears to have very little conservation across the species. There is an initial short sequence of conserved residues in the N-terminus of the tail that rapidly loses similarity, most notably in the rat isoform, which is massively truncated relative to the mouse and human isoforms. There are no conserved predicted phosphorylation sites, and the predicted secondary structure only suggests that there is almost no regular structure in any of the three species. The hydropathy profiles are also inconsistent. Although the rat isoform is truncated, it has a similar initial hydropathy profile to the mouse. The human isoform is similar in length to the mouse isoform, but does not share a similar hydropathy profile until after the first ∼20 residues. GFAPκ forms heteromeric structures with GFAPα [36], and the tail region of GFAP proteins is important to stabilization of the IF through lateral interactions in the protofibrils/protofilaments. The specific role of GFAPκ has not been determined, but the profile of the tail region suggests that it is not important to the role of GFAPκ, and that any conservation is coincidental. Alternatively, the shared similarities in the hydropathy profile of human and mouse (but not sequence conservation) may indicate a shared, conserved role that is taken over in rat by another (as yet undiscovered) isoform.

It is becoming increasingly evident that IFs are dynamic structures, with the ability to reorganize within a cell [55]. The GFAP isoform family represents an appropriate model for investigations of how modulations of cytoskeletal network participate in the regulation of cellular functions and interactions. With respect to the 3′ splice variants of GFAP, the tail region is important in filament diameter, and it is clear that the variations in the tail regions of GFAPδ/ε and GFAPκ must have an effect on filament formation. Analysis at the DNA level finds features that explain and define the 3′ GFAP splice variants, and then further analysis at the protein level reinforces these findings, and further defines characteristics of this isoform family. Several distinctive features are conserved across species, suggesting that they are fundamental to specific functions, rather than incidental or irrelevant variations in GFAP. The conserved variation at both the DNA and protein level for each of the individual isoforms is indicative of tight regulation of the generation of the isoforms, with a specific function for each that goes beyond structure regulation, and is important to the diversity of structure and function of astrocytes in homeostatic and disease states.

## Methods

### Multiple change-point analysis

A 46-way multiz alignment was obtained from UCSC using human as the reference species, for a 110 Kb genomic region spanning the GFAP gene and flanked by ∼50 Kb upstream and downstream (coordinates human chr17:42933000–43043000). Assemblies in the multiz alignment were hg19, mm9 and rn4. The GALAXY server (http://main.g2.bx.psu.edu/) was used to extract the three species from each multiz alignment, and convert the maf format to fasta. Note that the change-point method is not limited to 3 species - our current implementation can be used with up to 10 aligned species, and in principle could be modified to allow for even more. The method has not been applied to more than four aligned species, but we do not anticipate that a larger number would pose any difficulty. In principle, adding species should enable finer distinctions to be made between classes of conservation levels, so that the classes identified below could be differentiated into sub-classes.

For each species, a three-character conservation code (0, 1 or 2) was generated for each column of the respective alignment, where a ‘0’ was used when all the nucleotides at the given position were the same, ‘1’ was used when there were two distinct nucleotides at that position, and ‘2’ was used in positions where there were three different nucleotides. Indels were treated as an extra character, i.e. a single change. Runs of indels were treated as consecutive characters, not compressed into a single character as we have done in other studies. This may introduce some Markov dependence between adjacent characters of the sequence, and the model does not take this dependence into account. However, we reasoned that this mismatch between the model and the data would be of less significance than retaining information about the conservation of individual bases, which would be lost if runs of indels were compressed. Such information is important for resolving distinct classes of conservation level. Segmentation analysis was performed using the changept software using from 1–10 classes of segment [47], [48]. Selecting the appropriate number of classes is a difficult problem, to which no entirely satisfactory solution exists. We selected the 4-class model using information criteria described in [74]. The values of the approximations to AIC, BIC and DIC are plotted in Figure S8 for each number of classes. DIC is apparently too variable to be useful in this analysis, and BIC is also displaying unusual behaviour, in that it increases with the number of classes. This leaves AIC, which has achieved most of its reduction in value at four classes, with additional classes providing comparatively little improvement. Four profiles showing the posterior probability that each sequence position belongs to each of the four classes were generated and converted to wiggle tracks for viewing in the UCSC browser (Figure 2).

Convergence characteristics of the MCMC sampler for 4 classes are discussed in Supporting Information S1.

To calculate the sensitivity and specificity of the Group 2 profile considered as a classifier for protein-coding sequence, positions in known protein-coding sequence with a Group 2 profile greater than 0.5 were classed as true positives (TP), or otherwise classed as false negatives (FN). Non-protein-coding positions (including UTRs) with a Group 2 profile greater than 0.5 were classed as true negatives (TN), and otherwise classed as false positives (FP). Sensitivity = TP/(TP+FN) = 0.867, specificity = TN/(TN+FP) = 0.937. Note that these values are only poor approximations because there may be additional unidentified protein-coding sequence in GFAP. Note also that the non-protein coding sequence of GFAP also contains conserved features other than protein-coding sequence, in particular those highlighted in Figure 3, especially in Features A and C, and the Features E and F.

### GFAP protein sequences

Sequences were obtained from the NCBI protein database (http://www.ncbi.nlm.nih.gov/protein/) for GFAPα, GFAPδ/ε and GFAPκ with the following reference numbers. Human: NP_002046.1 (GFAPα), NP_001124491.1 (GFAPδ/ε), ABL14186.1 (GFAPκ). Mouse: P03995.4 (GFAP α), NP_001124492.1 (GFAPδ/ε). Rat: AAD01873.1 (GFAPα), AAD01874.2 (GFAPδ/ε), ABL14185.1 (GFAPκ). Mouse GFAPκ was reconstructed from the genomic sequence, and cross-referenced with the sequence reported in [36].

## Supporting Information

Figure S1 **The proportions of the 10 possible alignment patterns for each of the 4 classes.** ‘AAA’ represents an alignment column with three matching bases. ‘AAB’ indicates an alignment column in which human matches mouse but differs from rat, and similarly for ‘ABA’ and ‘ABB’. ‘ABC’ represents an alignment column in which all three species have different bases. The remaining 5 codes represent alignment columns containing one or two indel characters ‘-’. Note that the alignment does not include any indels in the human sequence, as human is used as the reference species.
(PDF)

Figure S2 **Comparison of the four class profiles for conserved feature B.** Conserved feature B is not in fact unambiguously assigned to Group 2 (the conserved class), but has a slightly elevated Group 2 profile relative to the surrounding sequence. The four profiles are shown in one plot for comparison.
(PDF)

Figure S3 **The proportions of the 10 possible alignment patterns in Feature B, Class 2 and Class 3.** Data were evaluated as described in Figure S1.
(PDF)

Figure S4 **Comparison of the four class profiles for conserved feature D.** Conserved feature D is not unambiguously assigned to Group 2 (the conserved class), but has a slightly elevated Group 2 profile relative to the surrounding sequence. Figure S4 shows the four profiles in one plot for comparison.
(PDF)

Figure S5 **The proportions of the 10 possible alignment patterns in Feature D, Class 2 and Class 3.** Data were evaluated as described in Figure S1.
(PDF)

Figure S6 **TOMTOM output indicating a possible match of the EBF1 transcription factor binding motif in conserved feature B.** The human DNA sequence of conserved Feature B (Figure 3) is: GCAAAGGGATCCAGCTCTCCCTGGGGGCCTTCGTGACAC. This sequence was compared against known motifs using the TOMTOM database (http://meme.sdsc.edu/meme/cgi-bin/tomtom.cgi) with the default options (IUPAC query motif, JASPAR and UNIPROBE databases). One significant match was detected, with p-value 6.7×10^−6^ and E-value 0.0058. The match indicates a putative binding site for the transcription factor early B-cell factor 1 (EBF1). However, no such putative binding site was identified in the mouse or rat sequences when they were similarly analyzed, and hence the match may be spurious. A screen shot of the output is included here.
(PDF)

Figure S7 **Values of AIC, BIC and DIC for independent MCMC runs with 1 to 12 classes of segment.** A 464 nt human sequence spanning GFAP exons 7 and 7a was submitted to the Human Splicing Finder server (http://www.umd.be/HSF/HSF.html). A screen shot of the output is included below. Exon 7a is not annotated in HSF, but begins at position +354 (not shown, but at the right hand end of the red horizontal bar in the figure below). The likely natural acceptor site (which is annotated by HSF as potential) is indicated. The red horizontal bar indicates the approximate location of the group 2 sequence (“conserved” sequence) upstream of 7a (note that conserved feature C actually extends beyond the right hand end of the red horizontal bar, and covers Exon 7a). A potential acceptor site (score 76.63 out of a possible 100) is located 40 nt upstream of this conserved region, which is consistent with the hypothesis that the red horizontal bar includes a novel splice variant.
(PDF)

Figure S8 **Selection of the number of classes.** To identify the number of distinct classes of conservation pattern in the DNA sequence of GFAP, we used approximations to the well-known information criteria AIC, BIC and DIC. These approximations are discussed in [74]. Note that a lower value of the information criteria indicates a better model. Values of the three information criteria were determined for independent MCMC runs with the number of classes varying from 1 to 12. The results are shown in the plot below. We judged that DIC was too variable to be useful, and that BIC favoured a 1-class model, which is inappropriate. We therefore based our judgment on AIC, which indicates that most of the reduction in AIC occurs to the left of the 4-class model in the plot.
(PDF)

Supplementary Information S1 **Convergence to the posterior distribution.** In any MCMC analysis, it is important to check that convergence to the limiting distribution has occurred, and identify the length of the ‘burn-in’ period. This is most commonly assessed by inspecting a time-series plot of the log-likelihood. Firstly, we generated a plot for the 1000 iterations of the 4-class model, alongside a density plot for the same 1000 log-likelihood values. It is already clear from these plots that convergence occurred rapidly, certainly within the first 100 iterations. However, as an added check that all parameters of the model have converged, we plotted time-series for the following parameters, including data points from all 1000 iterations: 1. number of changepoints: the number of changepoints identified in the 100 kb genomic sequence; 2. mixture proportions (pi values): the proportion of segments assigned to each of the 4 groups; and 3. alpha 1, alpha 2, alpha 3: the parameters of the Dirichlet distribution of the proportions of conservation codes (i.e. 0, 1 and 2) in each group. * Note that for the alpha values for group 4, the values at iteration 994 are omitted for clarity in the plots: alpha 1 = 114451.3, alpha 2 = 16631.34, alpha 3 = 937.327. All of the parameters appear to have converged to a limiting distribution within 100 iterations. We therefore selected a ‘burn-in’ period of 500 iterations, and used only the last 500 samples in all subsequent analyses.
(PDF)

Table S1 **Summary statistics for the 4 classes.** To investigate the characteristics of the 4 classes identified by changept, we first identified the columns of the alignment that could be unambiguously assigned to each class. For example, an alignment column can be unambiguously assigned to Group 1 if the Group 1 profile value (the probability that the column belongs to Group 1) is greater than 0.5. The total number of alignment columns that could be unambiguously assigned to one of the four groups was 109,662 out of a total of 110,001 columns. For each class, we then counted the number of times each of 10 possible alignment column patterns occurred. In these patterns, ‘A’, ‘B’ and ‘C’ represent different nucleotides, such that ‘AAA’ is three identical nucleotides, and ‘ABC’ is three distinct nucleotides. The ‘-’ character represents an indel. The counts and corresponding proportions are given in the following table, and the proportions are displayed in Figure S1. The ten patterns are defined in the legend to Figure S1.
(PDF)

Table S2 **Comparison of the four class profiles for conserved feature B.** The maximum of the Group 2 profile for this feature occurs at position 42987863, where the four profile values are as shown.
(PDF)

Table S3 **Summary statistics for feature B, positions 42987853–42987890.** To investigate why there is an increased Group 2 profile in this region, we tabulated the proportions of each of the positions in feature B as follows, and we compared plots of the resulting distribution to similar plots obtained for Groups 2 and 3 in Supplementary Section 1. Note that the proportion of exactly matching columns (AAA) in feature B is actually more similar to that of Group 2 than Group 3.
(PDF)

Table S4 **Comparison of the four class profiles for conserved feature D.** The maximum of the Group 2 profile for this feature occurs at position 42987412, where the four profile values are as shown.
(PDF)

Table S5 **Summary statistics for feature D, positions 42987395–42987420.** To investigate why there is an increased Group 2 profile in this region, we tabulated the proportions of each of the positions in feature D as follows, and we compare plots of the resulting distribution to similar plots obtained for Groups 2 and 3 in Supplementary Section 1. Note that the proportion of exactly matching columns (AAA) in feature D is actually more similar to that of Group 2 than Group 3.
(PDF)
