# Supplementary figures and images for: Computational Characterization of 3′ Splice Variants in the GFAP Isoform Family
Source: PLoS One. 2012 Mar 30;7(3):e33565. doi: 10.1371/journal.pone.0033565 (PMC3316583; doi:10.1371/journal.pone.0033565)

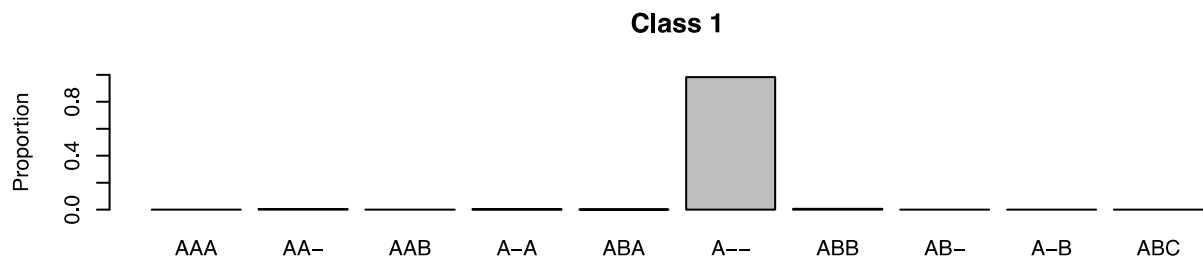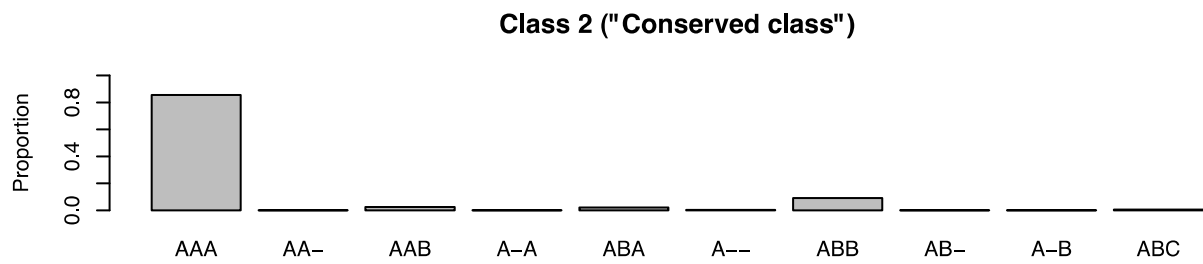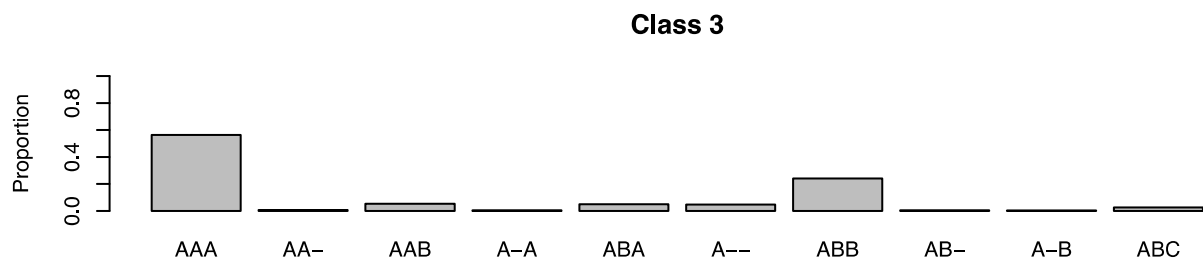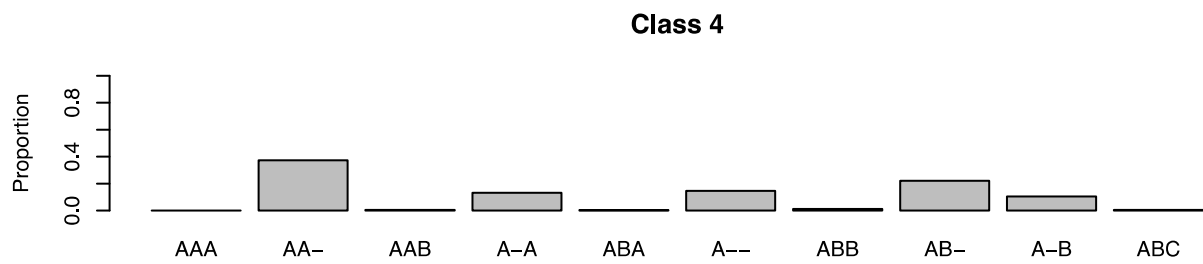

Supplement: Figure S1 — The proportions of the 10 possible alignment patterns for each of the 4 classes. ‘AAA’ represents an alignment column with three matching bases. ‘AAB’ indicates an alignment column in which human matches mouse but differs from rat, and similarly for ‘ABA’ and ‘ABB’. ‘ABC’ represents an alignment column in which all three species have different bases. The remaining 5 codes represent alignment columns containing one or two indel characters ‘-’. Note that the alignment does not include any indels in the human sequence, as human is used as the reference species. (PDF) [file pone.0033565.s001.pdf]

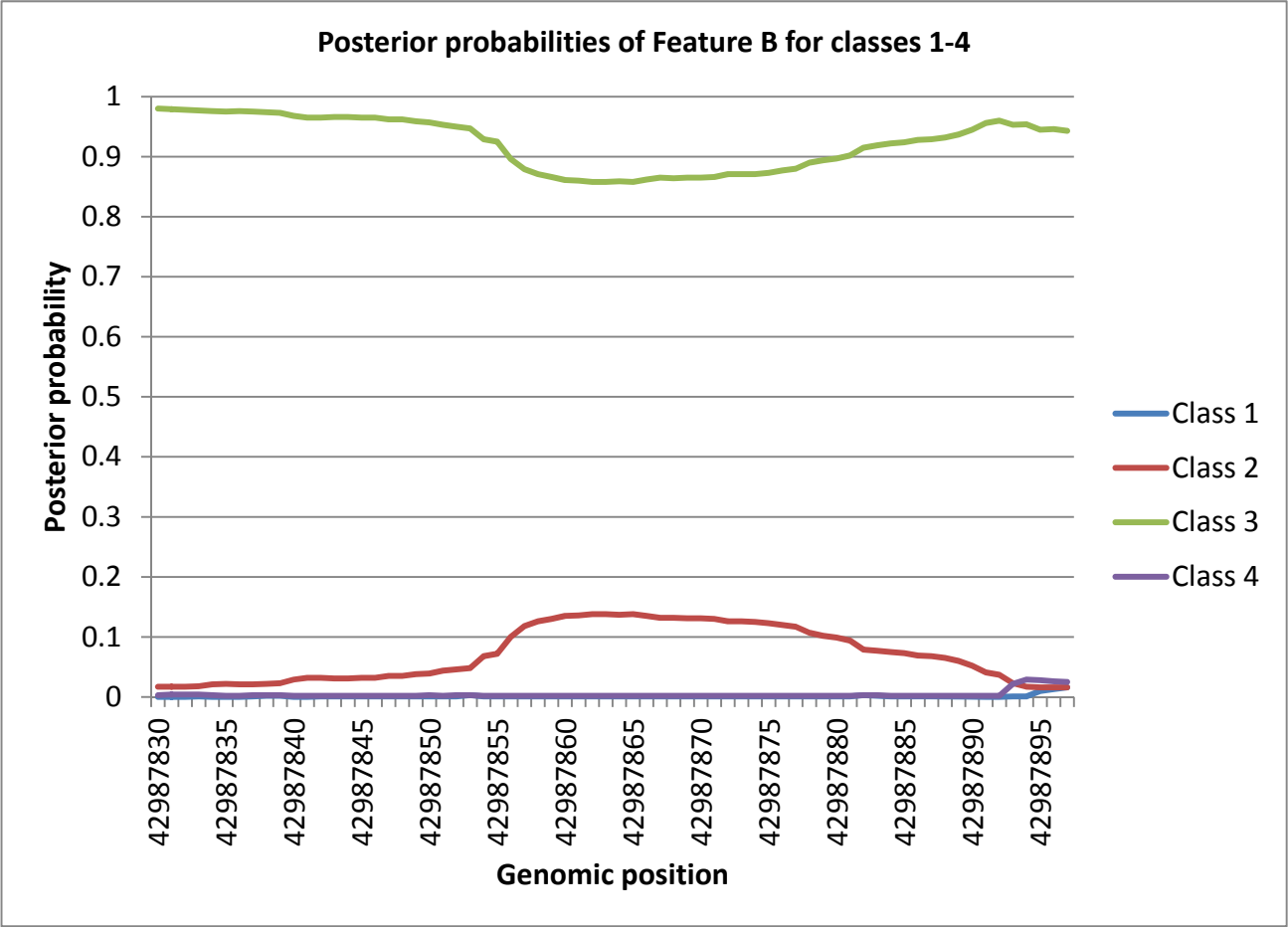

Supplement: Figure S2 — Comparison of the four class profiles for conserved feature B. Conserved feature B is not in fact unambiguously assigned to Group 2 (the conserved class), but has a slightly elevated Group 2 profile relative to the surrounding sequence. The four profiles are shown in one plot for comparison. (PDF) [file pone.0033565.s002.pdf]

**Feature B (42987853 – 42987890)**

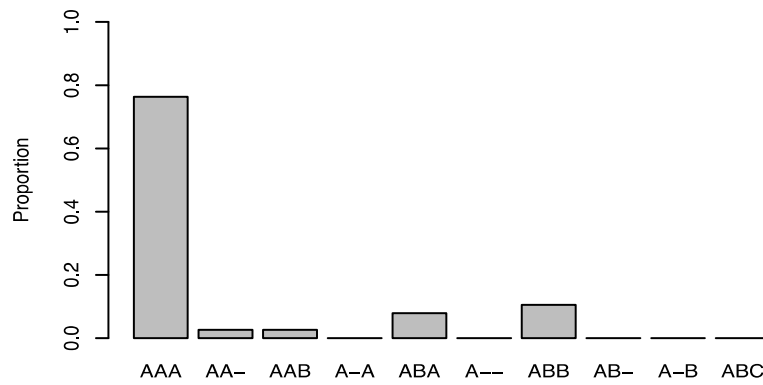

**Class 2 ("Conserved class")**

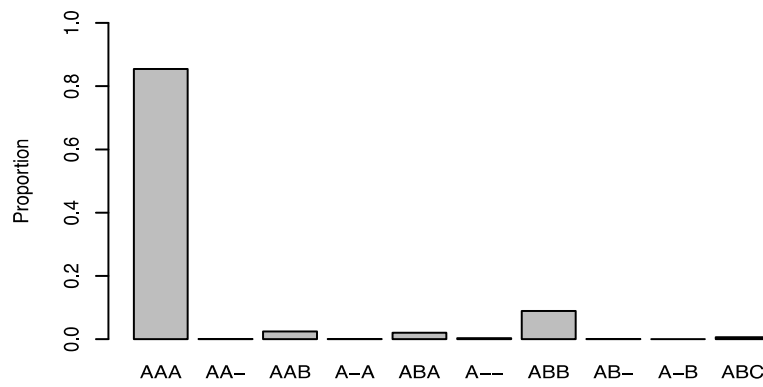

**Class 3**

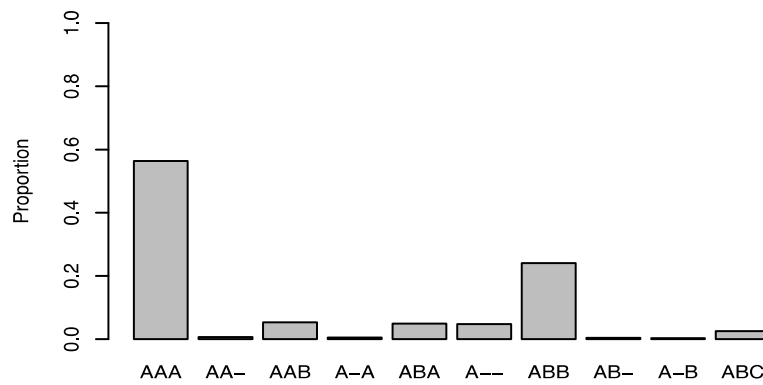

Supplement: Figure S3 — The proportions of the 10 possible alignment patterns in Feature B, Class 2 and Class 3. Data were evaluated as described in Figure S1. (PDF) [file pone.0033565.s003.pdf]

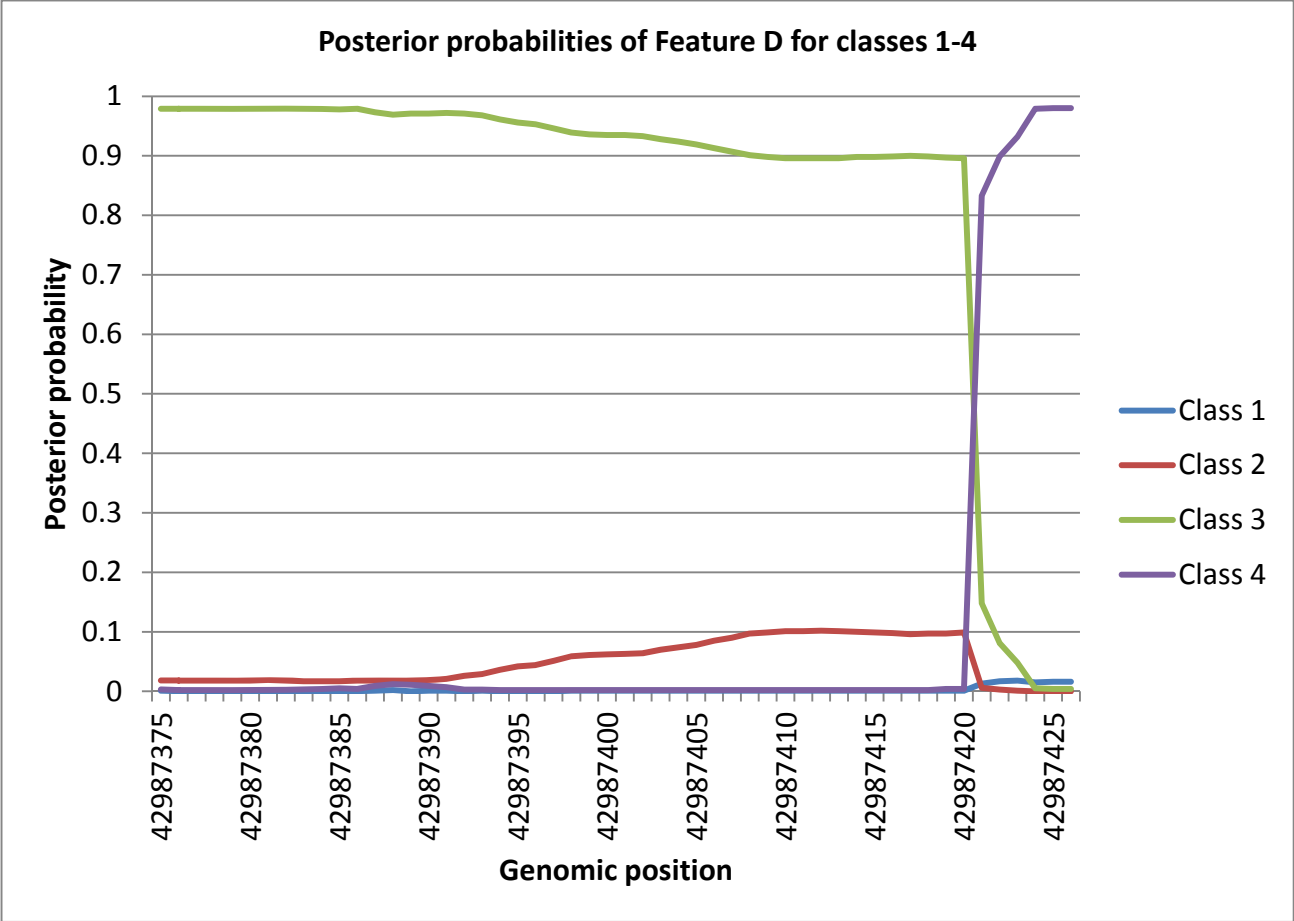

Supplement: Figure S4 — Comparison of the four class profiles for conserved feature D. Conserved feature D is not unambiguously assigned to Group 2 (the conserved class), but has a slightly elevated Group 2 profile relative to the surrounding sequence. Figure S4 shows the four profiles in one plot for comparison. (PDF) [file pone.0033565.s004.pdf]

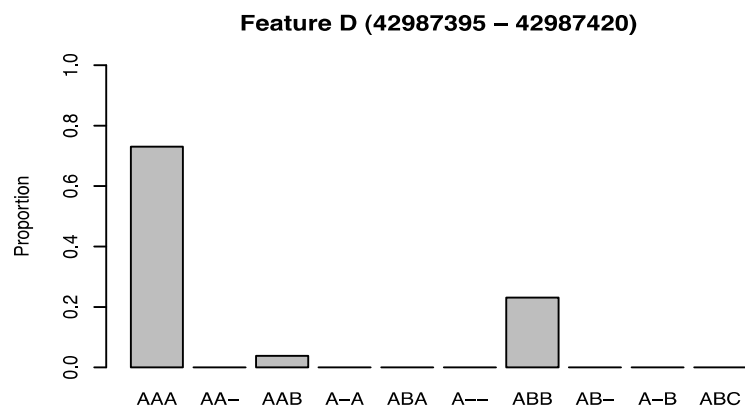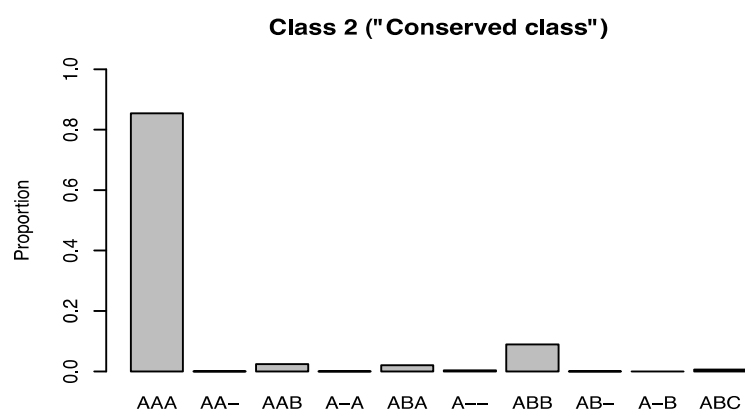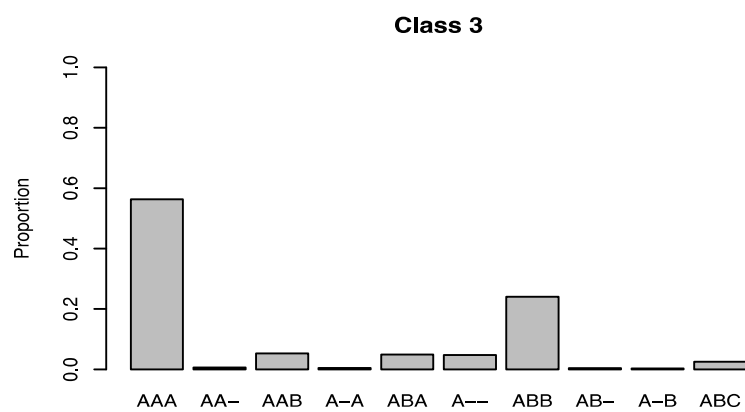

Supplement: Figure S5 — The proportions of the 10 possible alignment patterns in Feature D, Class 2 and Class 3. Data were evaluated as described in Figure S1. (PDF) [file pone.0033565.s005.pdf]

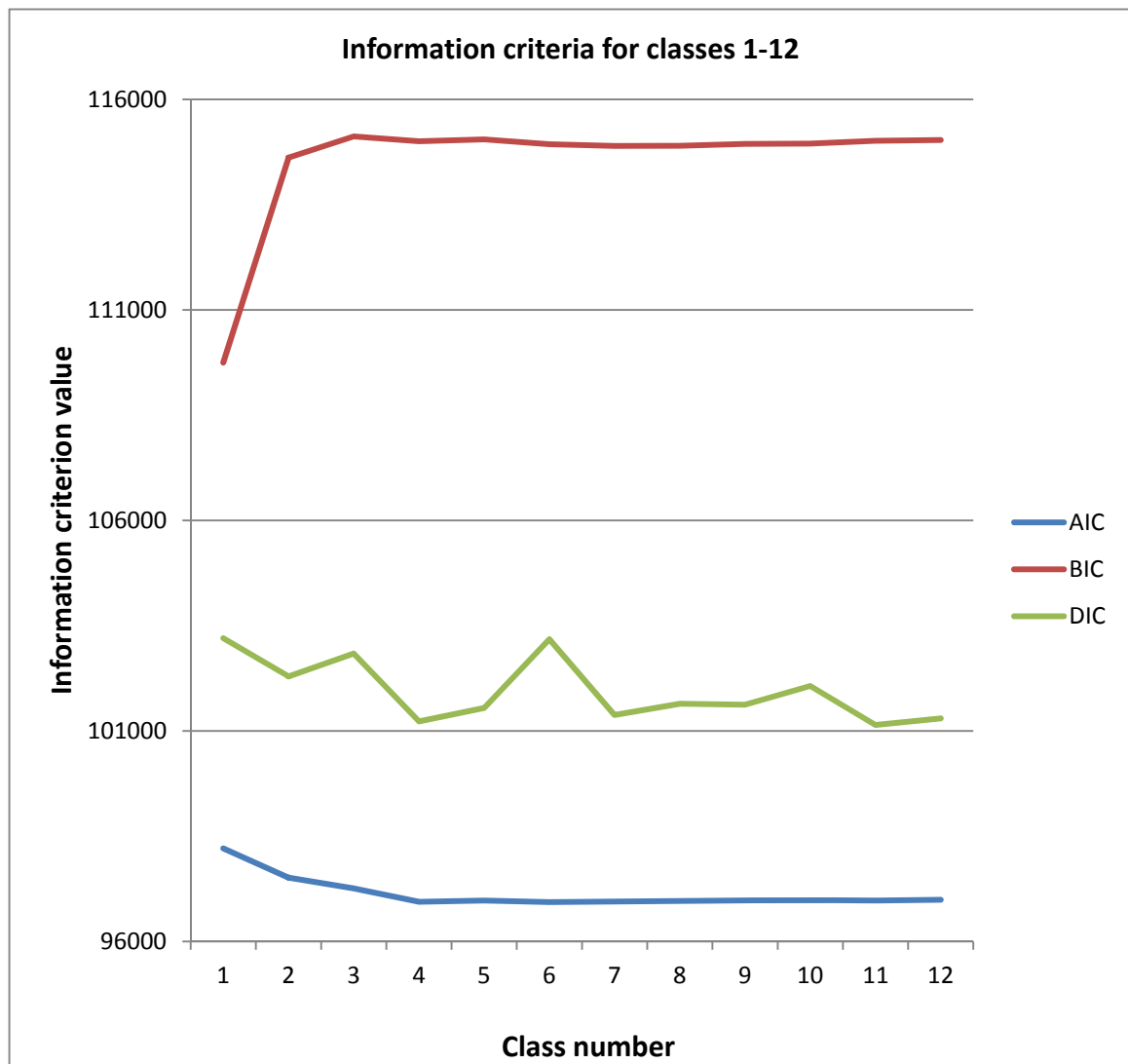

Supplement: Figure S8 — Selection of the number of classes. To identify the number of distinct classes of conservation pattern in the DNA sequence of GFAP, we used approximations to the well-known information criteria AIC, BIC and DIC. These approximations are discussed in [74]. Note that a lower value of the information criteria indicates a better model. Values of the three information criteria were determined for independent MCMC runs with the number of classes varying from 1 to 12. The results are shown in the plot below. We judged that DIC was too variable to be useful, and that BIC favoured a 1-class model, which is inappropriate. We therefore based our judgment on AIC, which indicates that most of the reduction in AIC occurs to the left of the 4-class model in the plot. (PDF) [file pone.0033565.s008.pdf]
