# Supplementary material for: Computational Characterization of 3′ Splice Variants in the GFAP Isoform Family
Source: PLoS One. 2012 Mar 30;7(3):e33565. doi: 10.1371/journal.pone.0033565 (PMC3316583; doi:10.1371/journal.pone.0033565)

**Name**	MA0154.1
**Alt. Name**	EBF1
**Database**	JASPAR_CORE_2009.meme
***p*-value**	6.72909e-06
***E*-value**	0.00580047
***q*-value**	0.0115713
**Overlap**	10
**Offset**	-17
**Orientation**	Reverse Complement

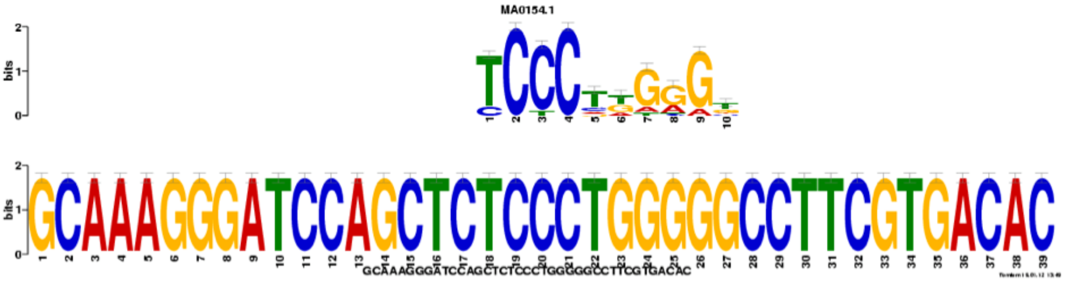

Supplement: Figure S6 — TOMTOM output indicating a possible match of the EBF1 transcription factor binding motif in conserved feature B. The human DNA sequence of conserved Feature B (Figure 3) is: GCAAAGGGATCCAGCTCTCCCTGGGGGCCTTCGTGACAC. This sequence was compared against known motifs using the TOMTOM database (http://meme.sdsc.edu/meme/cgi-bin/tomtom.cgi) with the default options (IUPAC query motif, JASPAR and UNIPROBE databases). One significant match was detected, with p-value 6.7×10^−6^ and E-value 0.0058. The match indicates a putative binding site for the transcription factor early B-cell factor 1 (EBF1). However, no such putative binding site was identified in the mouse or rat sequences when they were similarly analyzed, and hence the match may be spurious. A screen shot of the output is included here. (PDF) [file pone.0033565.s006.pdf]
