# Supplementary material for: Computational Characterization of 3′ Splice Variants in the GFAP Isoform Family
Source: PLoS One. 2012 Mar 30;7(3):e33565. doi: 10.1371/journal.pone.0033565 (PMC3316583; doi:10.1371/journal.pone.0033565)

Reference sequence

GFAP Gene > **ENST00000253408** Transcript (9 exons) > Exon number: **7** (44 bp) + **20** intronic nucleotides at 5' end and **400** at 3' end

```
  1 ctccctctgc tttctttcag GATCACCATT CCCGTGCAGA CCTTCTCCAA CCTGCAGATT CGAGgtcagt acagcagggc ctcgtgggaa gggcactgga
101 gtcctgcccc ctcctccagg gcctgtaggt tgctccagac tgggactgag gatcagggca aagggatcca gctctccctg ggggccttcg tgacactgca
201 gcgctcctag ccagagccta tcataccagg gtacttctag gtggggcttg cagctgcccc tgtcctgcta ggccctggtc cctcttcccc tccctgcacc
301 ccattcgaca gcagaactgg gtgagagctt gacatctgcc ctgtctgcag atccctgagc aagcactgcc cttctgagtg ttttctgttt ttgttttttt
401 aactgcttgt cactacaggg ggcaaaagca ccaaagacgg ggaaaatcac aaggtcacaa gata
```

Total sequence length: 464 nucleotides

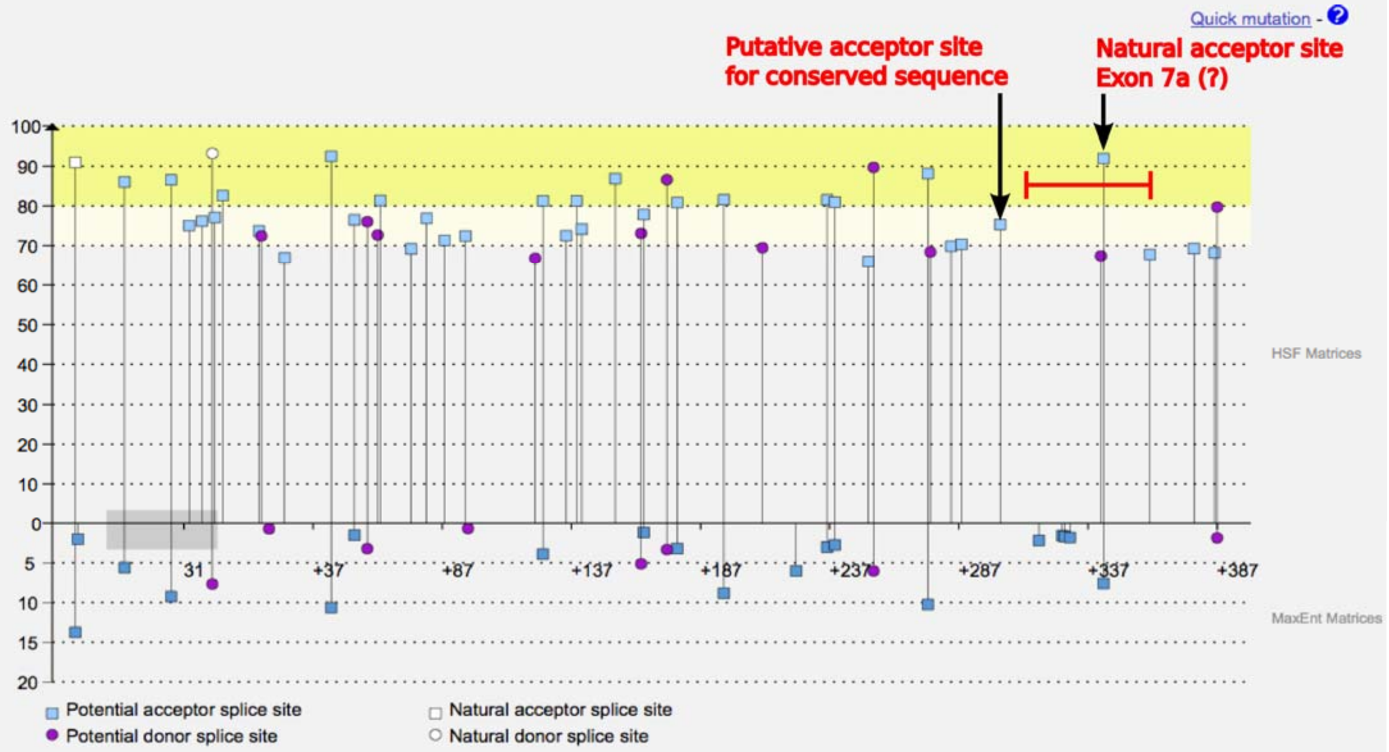

Supplement: Figure S7 — Values of AIC, BIC and DIC for independent MCMC runs with 1 to 12 classes of segment. A 464 nt human sequence spanning GFAP exons 7 and 7a was submitted to the Human Splicing Finder server (http://www.umd.be/HSF/HSF.html). A screen shot of the output is included below. Exon 7a is not annotated in HSF, but begins at position +354 (not shown, but at the right hand end of the red horizontal bar in the figure below). The likely natural acceptor site (which is annotated by HSF as potential) is indicated. The red horizontal bar indicates the approximate location of the group 2 sequence (“conserved” sequence) upstream of 7a (note that conserved feature C actually extends beyond the right hand end of the red horizontal bar, and covers Exon 7a). A potential acceptor site (score 76.63 out of a possible 100) is located 40 nt upstream of this conserved region, which is consistent with the hypothesis that the red horizontal bar includes a novel splice variant. (PDF) [file pone.0033565.s007.pdf]
