# Supplementary material for: Computational Characterization of 3′ Splice Variants in the GFAP Isoform Family
Source: PLoS One. 2012 Mar 30;7(3):e33565. doi: 10.1371/journal.pone.0033565 (PMC3316583; doi:10.1371/journal.pone.0033565)
Supplement: Supplementary Information S1 — Convergence to the posterior distribution. In any MCMC analysis, it is important to check that convergence to the limiting distribution has occurred, and identify the length of the ‘burn-in’ period. This is most commonly assessed by inspecting a time-series plot of the log-likelihood. Firstly, we generated a plot for the 1000 iterations of the 4-class model, alongside a density plot for the same 1000 log-likelihood values. It is already clear from these plots that convergence occurred rapidly, certainly within the first 100 iterations. However, as an added check that all parameters of the model have converged, we plotted time-series for the following parameters, including data points from all 1000 iterations: 1. number of changepoints: the number of changepoints identified in the 100 kb genomic sequence; 2. mixture proportions (pi values): the proportion of segments assigned to each of the 4 groups; and 3. alpha 1, alpha 2, alpha 3: the parameters of the Dirichlet distribution of the proportions of conservation codes (i.e. 0, 1 and 2) in each group. * Note that for the alpha values for group 4, the values at iteration 994 are omitted for clarity in the plots: alpha 1 = 114451.3, alpha 2 = 16631.34, alpha 3 = 937.327. All of the parameters appear to have converged to a limiting distribution within 100 iterations. We therefore selected a ‘burn-in’ period of 500 iterations, and used only the last 500 samples in all subsequent analyses. (PDF) [file pone.0033565.s009.pdf]

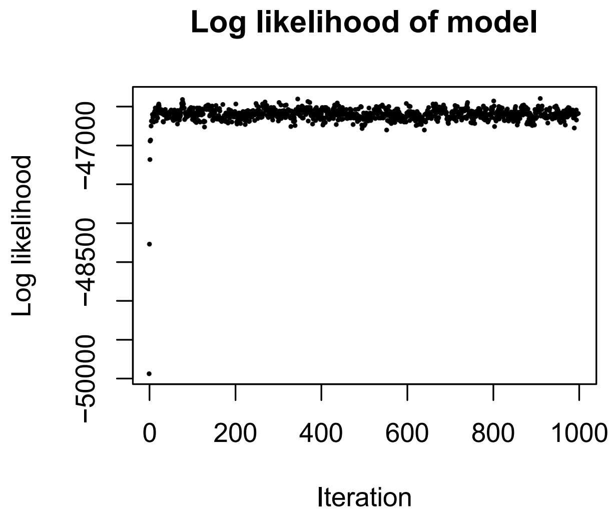
Log likelihood of model
Log likelihood
−47000
−48500
−50000
0
200
400
600
800
1000
Iteration

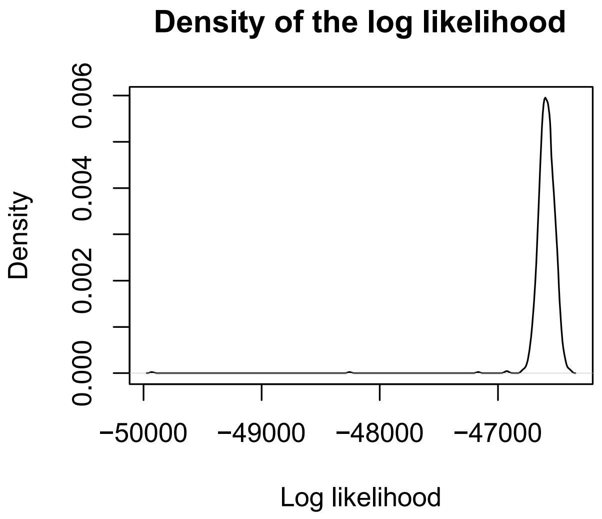
Density of the log likelihood
Density
0.000
0.002
0.004
0.006
−50000
−49000
−48000
−47000
Log likelihood

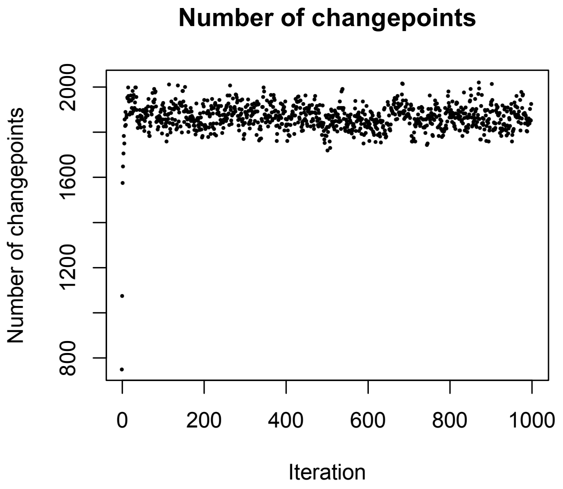
Number of changepoints
Number of changepoints
800
1200
1600
2000
0
200
400
600
800
1000
Iteration

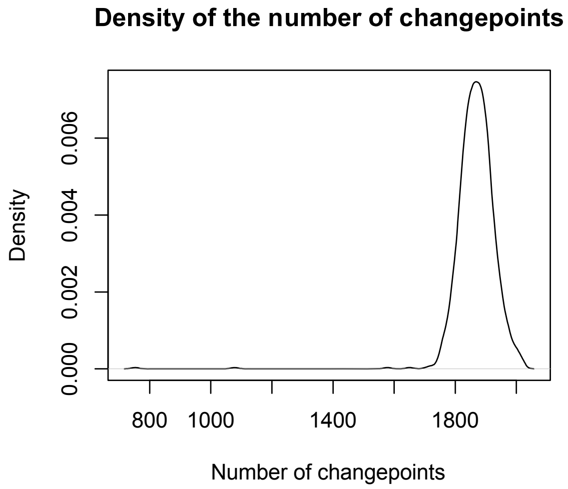
Density of the number of changepoints
Density
0.000
0.002
0.004
0.006
800
1000
1400
1800
Number of changepoints

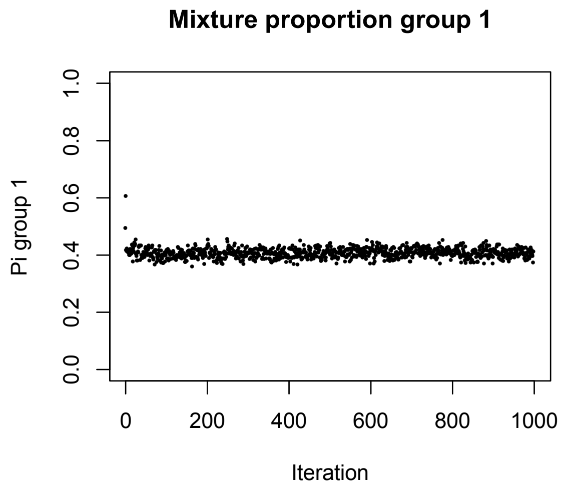
Mixture proportion group 1
Pi group 1
0.0
0.2
0.4
0.6
0.8
1.0
0
200
400
600
800
1000
Iteration

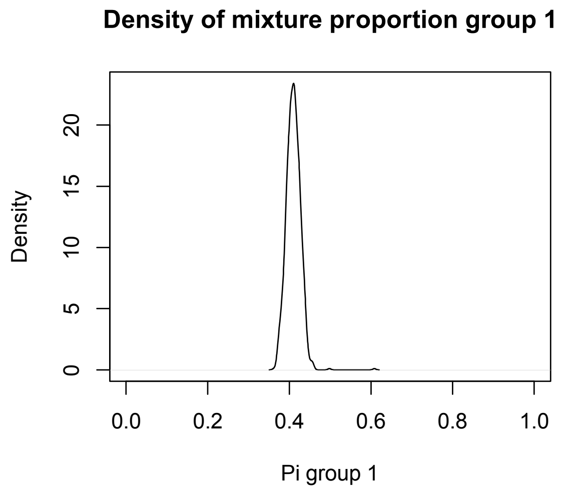
Density of mixture proportion group 1
Density
0
5
10
15
20
0.0
0.2
0.4
0.6
0.8
1.0
Pi group 1

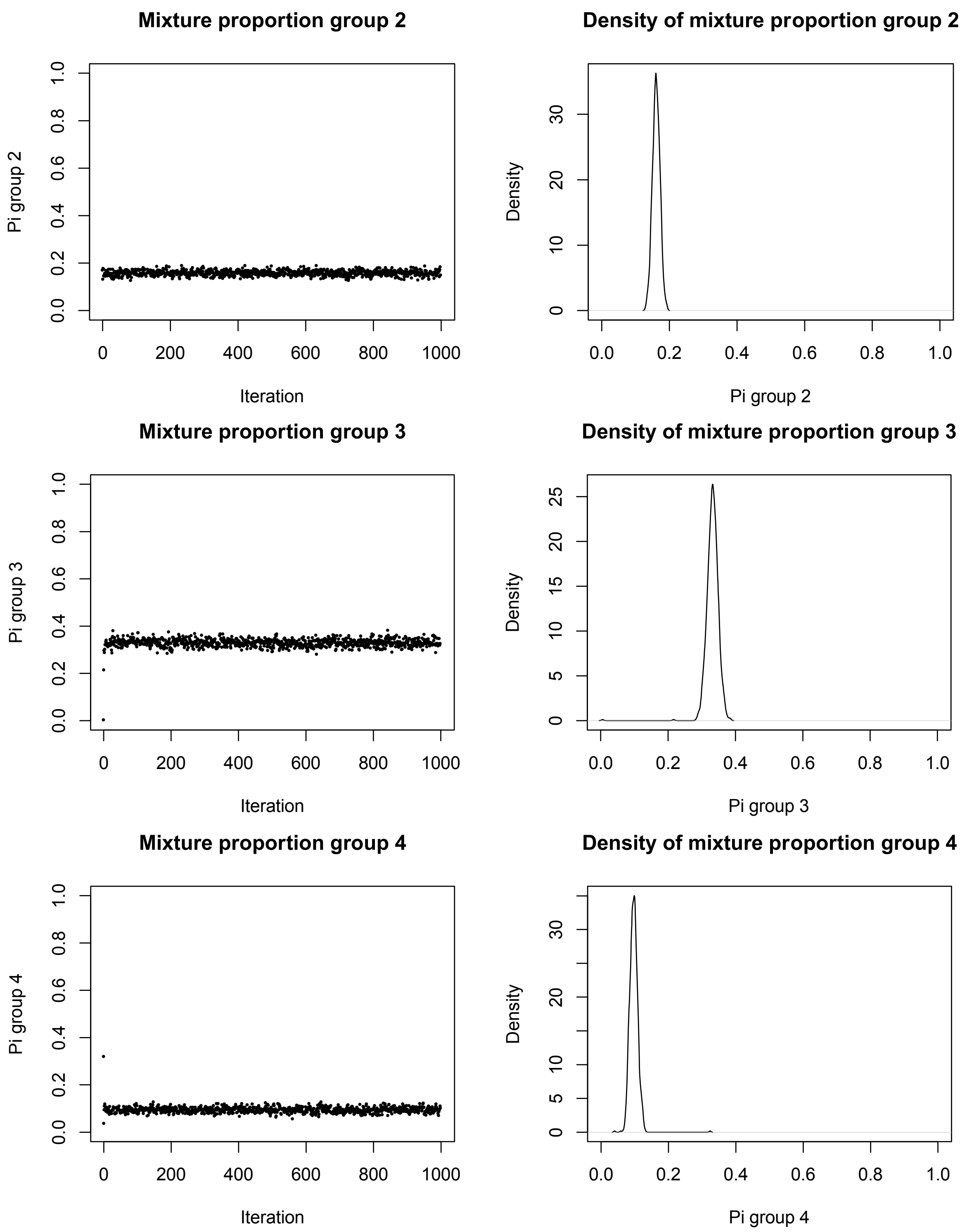
Mixture proportion group 2
Pi group 2
Iteration
0.0
0.2
0.4
0.6
0.8
1.0
0
200
400
600
800
1000
Density of mixture proportion group 2
Density
0
10
20
30
Pi group 2
Mixture proportion group 3
Pi group 3
Iteration
Density of mixture proportion group 3
Density
5
15
25
Pi group 3
Mixture proportion group 4
Pi group 4
Iteration
Density of mixture proportion group 4
Density
Pi group 4

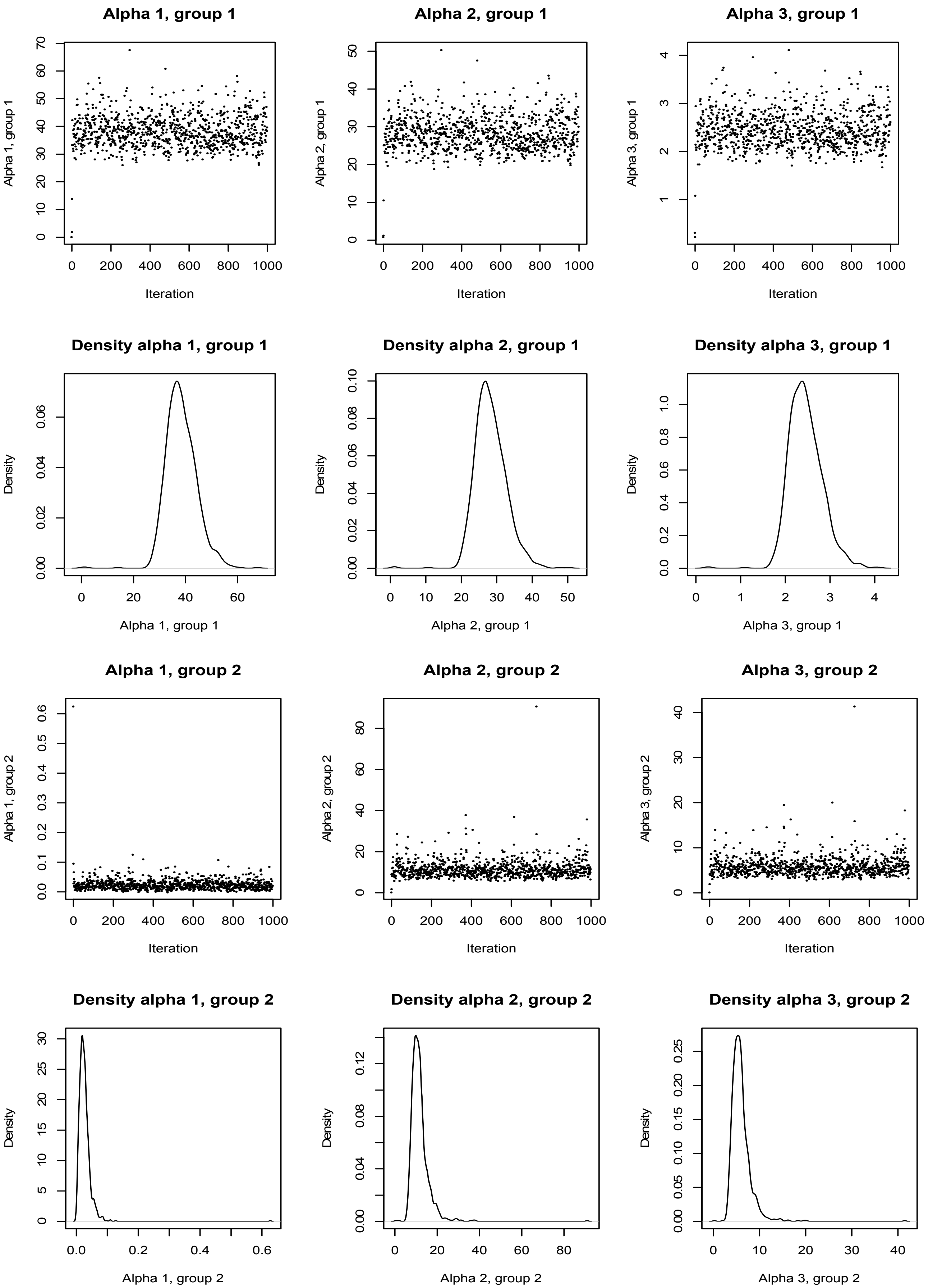
Alpha 1, group 1
Alpha 2, group 1
Alpha 3, group 1
Alpha 1, group 1
Alpha 2, group 1
Alpha 3, group 1
Iteration
Density alpha 1, group 1
Density alpha 2, group 1
Density alpha 3, group 1
Density
Alpha 1, group 1
Alpha 2, group 1
Alpha 3, group 1
Alpha 1, group 2
Alpha 2, group 2
Alpha 3, group 2
Iteration
Density alpha 1, group 2
Density alpha 2, group 2
Density alpha 3, group 2
Density
Alpha 1, group 2
Alpha 2, group 2
Alpha 3, group 2

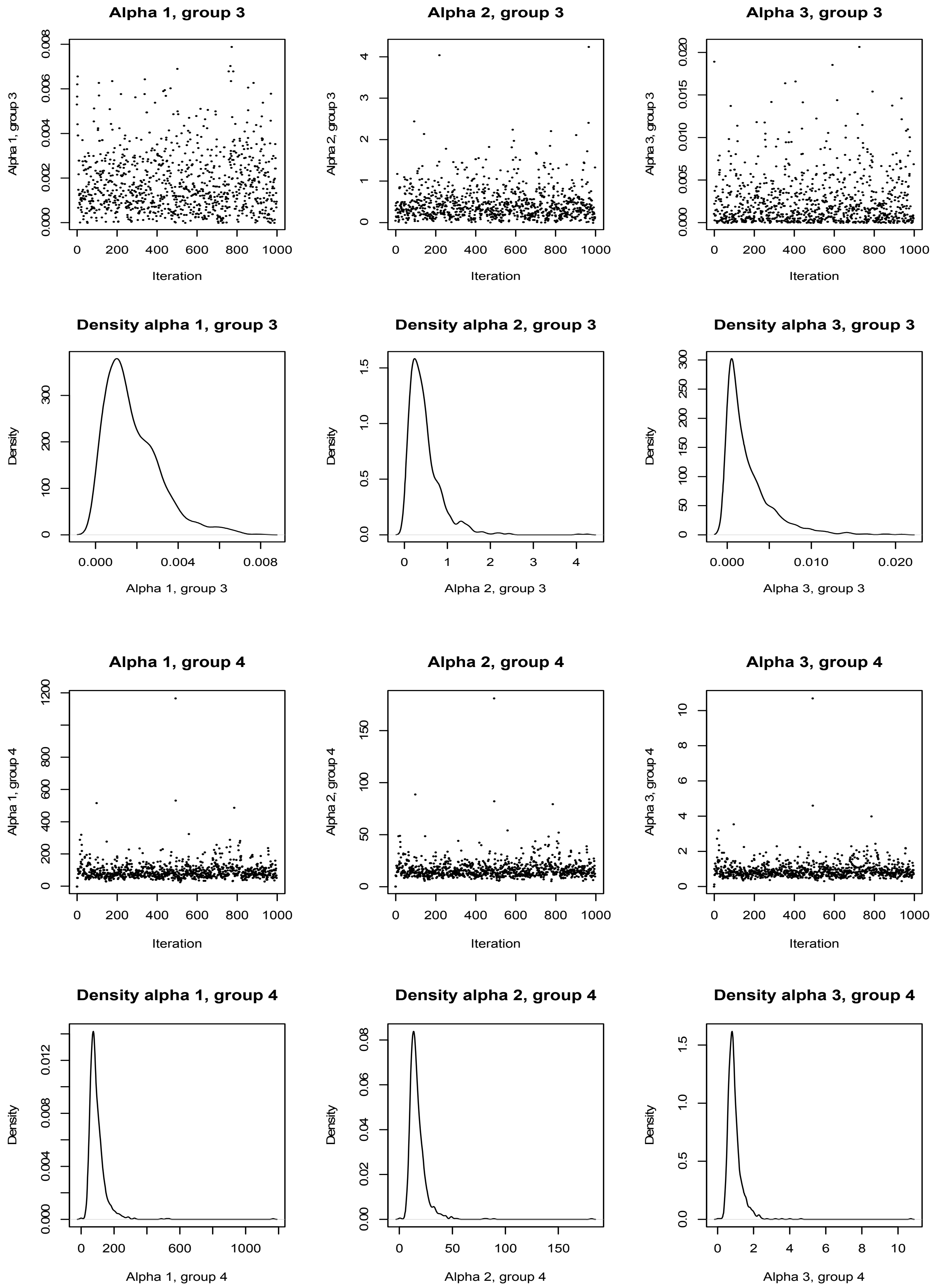
Alpha 1, group 3
Alpha 2, group 3
Alpha 3, group 3
Iteration
Density alpha 1, group 3
Density alpha 2, group 3
Density alpha 3, group 3
Density
Alpha 1, group 4
Alpha 2, group 4
Alpha 3, group 4
Iteration
Density alpha 1, group 4
Density alpha 2, group 4
Density alpha 3, group 4
Density
