# Supplementary material for: Computational Characterization of 3′ Splice Variants in the GFAP Isoform Family
Source: PLoS One. 2012 Mar 30;7(3):e33565. doi: 10.1371/journal.pone.0033565 (PMC3316583; doi:10.1371/journal.pone.0033565)
Supplement: Table S1 — Summary statistics for the 4 classes. To investigate the characteristics of the 4 classes identified by changept, we first identified the columns of the alignment that could be unambiguously assigned to each class. For example, an alignment column can be unambiguously assigned to Group 1 if the Group 1 profile value (the probability that the column belongs to Group 1) is greater than 0.5. The total number of alignment columns that could be unambiguously assigned to one of the four groups was 109,662 out of a total of 110,001 columns. For each class, we then counted the number of times each of 10 possible alignment column patterns occurred. In these patterns, ‘A’, ‘B’ and ‘C’ represent different nucleotides, such that ‘AAA’ is three identical nucleotides, and ‘ABC’ is three distinct nucleotides. The ‘-’ character represents an indel. The counts and corresponding proportions are given in the following table, and the proportions are displayed in Figure S1. The ten patterns are defined in the legend to Figure S1. (PDF) [file pone.0033565.s010.pdf]

	Group 1		Group 2		Group 3		Group 4	
Code	Total	Proportion	Total	Proportion	Total	Proportion	Total	Proportion
AAA	24	0.0005	10617	0.8546	22756	0.5635	6	0.0006
AA-	213	0.0045	6	0.0005	273	0.0068	3659	0.3727
AAB	54	0.0011	306	0.0246	2145	0.0531	42	0.0043
A-A	136	0.0029	4	0.0003	209	0.0052	1290	0.1314
ABA	63	0.0013	259	0.0208	1998	0.0495	35	0.0036
A--	46252	0.9832	38	0.0031	1928	0.0477	1433	0.1460
ABB	298	0.0063	1111	0.0894	9735	0.2411	119	0.0121
AB-	0	0.0000	5	0.0004	173	0.0043	2160	0.2200
A-B	0	0.0000	3	0.0002	133	0.0033	1026	0.1045
ABC	0	0.0000	75	0.0060	1031	0.0255	47	0.0048
	47040		12424		40381		9817	
