# Supplementary material for: Computational Characterization of 3′ Splice Variants in the GFAP Isoform Family
Source: PLoS One. 2012 Mar 30;7(3):e33565. doi: 10.1371/journal.pone.0033565 (PMC3316583; doi:10.1371/journal.pone.0033565)
Supplement: Table S2 — Comparison of the four class profiles for conserved feature B. The maximum of the Group 2 profile for this feature occurs at position 42987863, where the four profile values are as shown. (PDF) [file pone.0033565.s011.pdf]

Class 1	0.001
Class 2	0.138
Class 3	0.858
Class 4	0.002
