# Supplementary material for: Computational Characterization of 3′ Splice Variants in the GFAP Isoform Family
Source: PLoS One. 2012 Mar 30;7(3):e33565. doi: 10.1371/journal.pone.0033565 (PMC3316583; doi:10.1371/journal.pone.0033565)
Supplement: Table S3 — Summary statistics for feature B, positions 42987853–42987890. To investigate why there is an increased Group 2 profile in this region, we tabulated the proportions of each of the positions in feature B as follows, and we compared plots of the resulting distribution to similar plots obtained for Groups 2 and 3 in Supplementary Section 1. Note that the proportion of exactly matching columns (AAA) in feature B is actually more similar to that of Group 2 than Group 3. (PDF) [file pone.0033565.s012.pdf]

Code	Total	Proportion
AAA	29	0.7632
AA-	1	0.0263
AAB	1	0.0263
A-A	0	0.0000
ABA	3	0.0789
A--	0	0.0000
ABB	4	0.1053
AB-	0	0.0000
A-B	0	0.0000
ABC	0	0.0000
	38	
