# Supplementary material for: Computational Characterization of 3′ Splice Variants in the GFAP Isoform Family
Source: PLoS One. 2012 Mar 30;7(3):e33565. doi: 10.1371/journal.pone.0033565 (PMC3316583; doi:10.1371/journal.pone.0033565)
Supplement: Table S4 — Comparison of the four class profiles for conserved feature D. The maximum of the Group 2 profile for this feature occurs at position 42987412, where the four profile values are as shown. (PDF) [file pone.0033565.s013.pdf]

| | |
|---|---|
| Class 1 | 0.001 |
| Class 2 | 0.102 |
| Class 3 | 0.896 |
| Class 4 | 0.002 |
